# Updated Review on Natural Polyphenols: Molecular Mechanisms, Biological Effects, and Clinical Applications for Cancer Management

**DOI:** 10.3390/biom15050629

**Published:** 2025-04-28

**Authors:** Zainab Sabry Othman Ahmed, Elyas Khan, Nathan Elias, Alhussein Elshebiny, Qingping Dou

**Affiliations:** 1 Department of Cytology and Histology, Faculty of Veterinary Medicine, Cairo University, Giza 12211, Egypt; 2 Department of Anatomy and Histology, Faculty of Veterinary Medicine, King Salman International University, Ras Sudr 46612, Egypt; 3 Departments of Oncology, Pharmacology and Pathology School of Medicine, Barbara Ann Karmanos Cancer Institute, Wayne State University, Detroit, MI 48201, USA; elyaskhan882@gmail.com (E.K.); nathanelias7000@gmail.com (N.E.); alelshebi@gmail.com (A.E.)

**Keywords:** dietary sources, polyphenols, phenolic acids, anticancer, cell cycle arrest, apoptosis

## Abstract

Polyphenols, naturally occurring compounds found exclusively in plants, have gained significant attention for their potential in cancer prevention and treatment. These compounds are known for their antioxidant properties and are abundant in various plant-based foods, such as vegetables, fruits, grains, and beverages. Recent studies have highlighted the broad spectrum of health benefits of polyphenols, including their antiviral, anti-inflammatory, and anticancer properties. In addition, these naturally derived compounds are increasingly important for drug discovery due to their high molecular diversity and novel biofunctionalities. This review provides an in-depth analysis of the current research and knowledge on the potential use of dietary polyphenols as bioactive compounds for the prevention and treatment of various cancers. This review aims to provide valuable insights into the mechanisms underlying the anticancer properties of phenolic compounds in both laboratory and clinical settings. Furthermore, this review highlights the positive clinical outcomes associated with the use of polyphenols as anticancer agents and offers guidance for future research to advance this promising field.

## 1. Introduction

Cancer is a broad category of illnesses in which aberrant cells multiply uncontrollably, starting in nearly any body part and possibly moving to adjacent or distant regions. It is one of the leading causes of death globally, and its prevalence is continuously increasing. According to research by the World Health Organization (WHO), cancer is a major cause of mortality, contributing to almost 10 million deaths in 2020, or about one out of every six deaths.

Maintaining wellness and preventing illness requires a suitable diet and lifestyle. Polyphenols are naturally occurring chemicals produced exclusively by plants and possess potent antioxidant properties [1]. Owing to their prevalence in plant-based foods such as vegetables, fruits, and grains and their antioxidant action, polyphenols have been extensively researched in recent years as adjuvants in reducing the risk factors for debilitating diseases such as diabetes, cancer and cardiovascular disease (CVD) [2]. Examining the role of polyphenols in important signalling pathways could help clarify how a diet rich in polyphenols affects cancer outcomes [3].

Numerous studies have shown that the consumption of polyphenols yields various health benefits, including antiviral, antioxidant, anti-inflammatory, anti-thrombogenic, anti-allergic, antihyperlipidemic, anti-diabetic, anti-asthma, and anticancer effects [4]. Therefore, in response to the growing need for the development of new natural-based therapies, polyphenols have garnered significant scientific attention and have been the subject of substantial investigation in recent years [5].

These molecules exert anticancer effects by targeting different checkpoints in malignant cells and have a high specificity for inducing cell cycle arrest, autophagy, and apoptosis [6]. They exert these anticancer effects by inhibiting telomere expression, angiogenesis, and metastasis, in addition to lowering the expression of transcription factors that regulate the expression of cytoprotective genes, lowering p53 activation, reducing Bcl-2 expression and mitochondrial membrane potential, and decreasing the expression of HIF-1α while increasing cellular apoptosis via downregulation of p-Akt expression [7,8].

The objective of this review is to provide an overview of the current research and knowledge regarding the potential use of dietary polyphenols as naturally occurring bioactive compounds for the prevention and treatment of various malignancies. In addition to offering guidance for future research, this review also sheds light on the mechanisms underlying the possible anticancer properties of phenolic compounds in both clinical and laboratory contexts, as well as the ensuing positive clinical benefits of polyphenols as anticancer compounds.

## 2. Classification of Polyphenols

Natural polyphenols refer to a large group of phenylpropanoids synthesised by plants as secondary metabolites, ranging from small molecules to highly polymerised compounds, mostly in the form of glycosides. At least 10,000 distinct chemicals with one or more aromatic rings and one or more hydroxyl groups are collectively referred to as polyphenols [9]. Polyphenols can be divided into flavonoids and non-flavonoids. Flavonoids can exist as glycosides or aglycones despite their fundamental structures being aglycones (the non-sugar portion of the corresponding glycoside). Anthocyanins, flavonols, flavan-3-ols, flavones, isoflavones, flavanones, and stilbenes are examples of flavonoids typically found in food [10].

## 3. The Dietary Sources of Different Polyphenol Compounds

**Figure 1:**
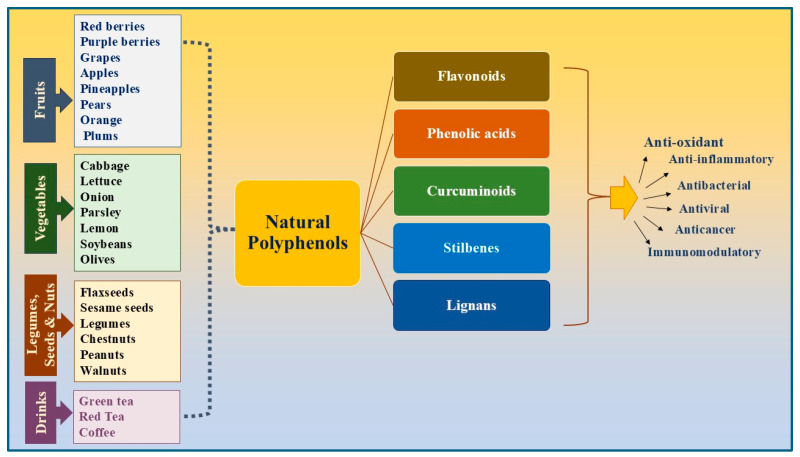
Dietary sources of natural polyphenols.

Most fruits and vegetables are rich in polyphenols. Certain fruits and vegetables contain higher levels of some polyphenols than others; therefore, identifying the primary dietary sources of each natural polyphenol is important. For example, sources of flavanols include onions and black tea. Moreover, flavanones are sourced from oranges and lemons. Epigallocatechin-3-gallate is predominantly found in green tea. Genistein is an isoflavone primarily found in soybeans. Quercetin is primarily found in onions, specifically red and yellow onions and citrus fruits. Phenolic acids, such as hydroxybenzoic acids, hydroxycinnamic acid, gallic acid, and caffeic acid, are found in nuts, pineapples, green tea, basil, olives, and other common natural substances. Curcumin, the most common source of curcuminoids, is found in turmeric. Resveratrol, a Stilbene, is primarily found in red grapes and red wine. Lignans, which are commonly found in flaxseeds, sesame seeds, and legumes, are present in a wide variety of foods. The general sources of these natural polyphenols (Figure 1) show that they can be easily and are most likely already incorporated into our daily diets.

## 4. Nutraceuticals and Pharmaceuticals Derived from Dietary Phenolic Compounds

High quantities of polyphenols have been partially linked to the anticancer properties of fruits and vegetables [11]. The well-studied polyphenols present in plants include stilbenes, lignans, phenolic acids, and flavonoids. The consumption of polyphenolic compounds has been linked to several health benefits (Table 1). Polyphenols influence cellular and molecular processes that impede several stages of carcinogenesis, including initiation, promotion, and progression [12]. The antiproliferative properties of polyphenols on various malignant tumours, both in vivo and in vitro, have been the subject of numerous investigations over the past few years [13]. These compounds selectively trigger cell cycle arrest, autophagy, and apoptosis and exert antiproliferative effects on a variety of human cancer cell types both in vivo and in vitro [6].

**Table 1:** Medicinal properties of natural phenolic compounds.

Class	Compound	Property(s)	Citation
1. Flavonoids	Anthocyanins	Antioxidant and Anticancer	[14]
	Flavanols	Anti-diabetic, Anti-inflammatory and Antioxidant	[15]
	Flavonols: Quercetin	Anti-inflammatory, Antioxidant, Antimicrobial, Anticancer, Antihypertensive, vasodilator, Antiobesity, Antiatherosclerosis	[16]
	Flavonols: Epigallocatechin-3-gallate	Antioxidant Anti-angiogenesis, Anti-inflammation and Anticancer	[17]
	Flavones; Luteolin	Anti-Inflammatory, Antioxidant, Antiallergy, Anticancer and Antibacterial	[18]
	Flavanones; Hesperetin	Anti-inflammatory, Antioxidant, Antibacterial and Anticancer	[18]
	Isoflavones; Genistein	Anti-inflammatory	[19]
2. Phenolic acids	Caffeic acid and its derivative caffeic acid phenethyl ester	Antioxidant, anti-inflammatory and anticancer	[20]
	Gallic acids	Anticancer, Antioxidant, and Anti-inflammatory	[21]
	Rosmarinic acid	Anti-inflammatory, Antiviral, Antibacterial, Antidepressant and Anticancer	[22]
	Sinapic acid	Antioxidant, Antimicrobial, Anti-inflammatory, Anticancer, Antianxiety	[23]
	Hydroxy benzoic acid	Antimicrobial	[24]
	Hydroxycinnamic acid	Antioxidant	[25]
	Protocatechuic acid	Anti-inflammatory and Antimicrobial	[26]
	Syringic acid	Antioxidant, antimicrobial, anti-inflammatory, antiendotoxic, neuro and hepatoprotective	[27]
	Protocatechoic acid	Anti-inflammatory and Antimicrobial	[26]
	Synergic acid	Antioxidant, antimicrobial, anti-inflammatory, antiendotoxic, neuro and hepatoprotective	[27]
	Vanillic acid	Antioxidant, Anti-inflammatory and Neuroprotective	[28]
3. Curcuminoids	Curcumin	Anti-inflammatory, Antioxidant and Anticancer	[29]
4. Stilbenes	Resveratrol	Antioxidant, Anti-inflammatory, Immunomodulatory, Neuroprotective, Cardiovascular protective and Anticancer	[30]
5. Lignans	Dibenzocyclooctadiene lignans	Antioxidant, Antiviral, Anti-inflammatory and Anticancer	[31]

## 5. Polyphenols and Their Anticancer Properties with Insights into Their Molecular Mechanisms, Preclinical Studies, and Clinical Applications

Naturally occurring anticancer chemicals found in dietary phenolic compounds provide a variety of treatment and preventive alternatives for different cancer types. As these compounds can target different checkpoints in malignant cells, investigating their mechanisms of action may increase the effectiveness of treatment [11].

### 5.1. Flavonoids 

#### 5.1.1. Anthocyanins

**Figure 2:**
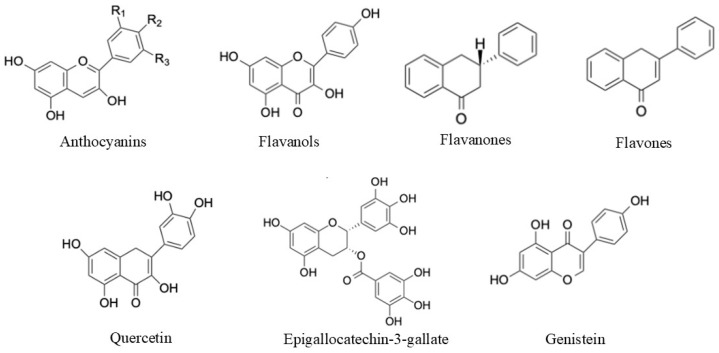
Chemical structures of different classes of flavonoids.

The most significant class of flavonoids found in plants is anthocyanins (Figure 2), which are water-soluble pigments that have shown antioxidant activity [32]. It has been reported that black elderberries, black chokeberries, and black currants are the richest sources of anthocyanins [33]. However, the bioavailability of anthocyanins is quite poor; only 1–2% of them retain their original structure after consumption [14]. PH, temperature, and solvents are some of the variables that affect the structures and characteristics of anthocyanins and should be managed when conducting investigations on their antioxidant activity [34].

The antimutagenic activity, suppression of oxidative DNA damage and carcinogens, cell cycle arrest, apoptosis, induction of phase II enzymes for detoxification, inhibition of cyclooxygenase-2 enzymes, and anti-angiogenesis are some of the potential mechanisms responsible for the anticancer activity of anthocyanins that have been described in various studies [35]. The downregulation of the pro-survival Sirt1/survivin and Akt/mTOR pathways, anti-proliferation, apoptosis, and decrease in the metastatic markers Sp1, Sp4, and VCAM-1 were confirmed in a variety of cell lines, including MDA-MB-231, MDA-MB-453, BT474, A17, N202/1A, and N202/1E [36]. Another study emphasised the induction of apoptosis via the p38/Fas/FasL/caspase eight and p38/p53/Bax signalling pathways [37]. Moreover, anthocyanins are known to exert strong anti-invasive and antimetastatic properties [14]. For instance, delphinidin treatment causes cell cycle arrest and apoptosis in several cancer types. By specifically inhibiting NF-κB-dependent MMP-9 (matrix metalloproteinase-9) gene expression, delphinidin can function as a potential antimetastatic drug that inhibits PMA-induced cancer cell invasion [38]. In addition, cyanidin-3-glucoside and cyanidin-3-rutinoside, which are extracted from mulberries, inhibited the migration and invasion of A549 lung cancer cells. Moreover, Peonidin-3-glucoside therapy also considerably inhibited lung cancer cell metastasis by downregulating matrix metalloproteinase (MMP) [39]. Furthermore, the growth and proliferation of 22Rv1, PC-3, and C4-2 prostate cancer cell lines were inhibited by anthocyanins [40]. Certain anthocyanins, such as cyanidin and delphinidin, have been shown to be cytotoxicity to colorectal cancer cells through oxidative stress [41].

Laboratory experiments using cell lines from several types of cancer (such as breast, colon, and prostate cancer) have revealed that anthocyanins can strongly inhibit the growth of cancer cells, in addition to inducing apoptosis. Additionally, anthocyanin supplementation decreased the size of tumours and suppressed tumour metastasis in animal experiments using mice [42]. Table 2 summarises the antitumor properties of anthocyanins reported in various malignancies in published studies.

**Table 2:** The anticancer properties of anthocyanins in different malignancies.

Anticancer Effect of Anthocyanins	Cancer Type	Citation
Anti-invasiveness and inhibition of the proliferation of MDA-MB-231 breast cancer cell lines.	Breast cancer	[43]
Reduction of the viability of breast cancer cell lines MCF-7, MDA-MB-231, and MDA-MB-453, in addition to induction of apoptosis in MDA-MB-453 cells via the intrinsic pathway (caspase cascade activation PARP cleavage and cytochrome C release) and suppression of tumour growth and angiogenesis via inhibiting MMP-9, MMP-2, and uPA expression in BALB/c naked mice with MDA-MB-453 cell xenografts.	Breast cancer	[44]
Inhibition of c-Jun N-terminal kinase, mitogen-activated protein kinase, and fibrosarcoma activation, downregulation of matrix metalloproteinase 2 secretion, and inhibition of cell migration and invasion in MDA-MB-453 breast cancer cells (HER2+).	Breast cancer	[45]
Inhibition of the development of abnormal crypt foci of colon in CF-1 mice.	Colon cancer	[46]
Induction of apoptosis in benign prostatic hyperplasia in rats.	Benign prostate hyperplasia	[47]
Triggering apoptotic factors such as TRAIL in cancer systems and suppression of Akt-mTOR signalling leading to maturation of acute myeloid leukaemia cells.	Leukaemia	[48]

#### 5.1.2. Flavanols

Dark chocolate and cocoa are the primary sources of flavanols [49], which are also found in berries, black chokeberries, blueberries, and blackcurrants. Other significant sources include strawberries, apples, hazelnuts, pecan nuts, pistachios, almonds, red wine, green tea, and black tea [50]. The positive outcomes were mostly linked to monomers/epicatechin/catechin and dimers/procyanidin B2/procyanidin. The bioavailability of procyanidins is approximately 100 times lower than that of their monomers. The monomers created following stomach breakdown that can be quickly absorbed in the gut are typically responsible for the biological effects. The metabolite production process, which is also attributed to the gut microbiota, may have a variety of biological impacts [10]. Isorhamnetin, a derivative of quercetin, exhibits impressive pharmacological properties, such as antioxidant and anti-inflammatory properties [51]. Moreover, the antioxidant properties of epicatechin (EPI), a naturally occurring flavonol, may facilitate the positive effects of natural products like cocoa [52].

#### 5.1.3. Flavonols

Flavonols are mostly found in fruits and vegetables such as cranberries and onions and in some drinks (such as tea and red wine), for which the estimated daily intake ranges from 18 (USA) to 58 mg (Japan) [53]. Nevertheless, these consumption thresholds often only address three main flavonols: kaempferol, myricetin, and quercetin [54]. Studies have investigated the health benefits of flavonol intake, with a focus on antioxidant activity, inflammatory biomarkers, and CVD risk factor reduction, in addition to the effect of quercetin, which has been enzymatically modified, on cognitive function [55].

##### Quercetin

Quercetin (3,3′,4′,5,7-Pentahydroxyflavone) (Figure 2) is a flavonol [54] with a variety of therapeutic uses [56,57], including cardiovascular protection and antiviral, anti-inflammatory, anti-allergic, and anticancer properties. Additionally, it has been discovered that quercetin is essential for plants [58] as it contributes to photosynthesis, growth, and seed germination due to its antibacterial and antioxidant properties. Moreover, quercetin’s presence in different brain regions aids in protection against several neurological disorders, including Parkinson’s and Alzheimer’s diseases [59].

**Figure 3:**
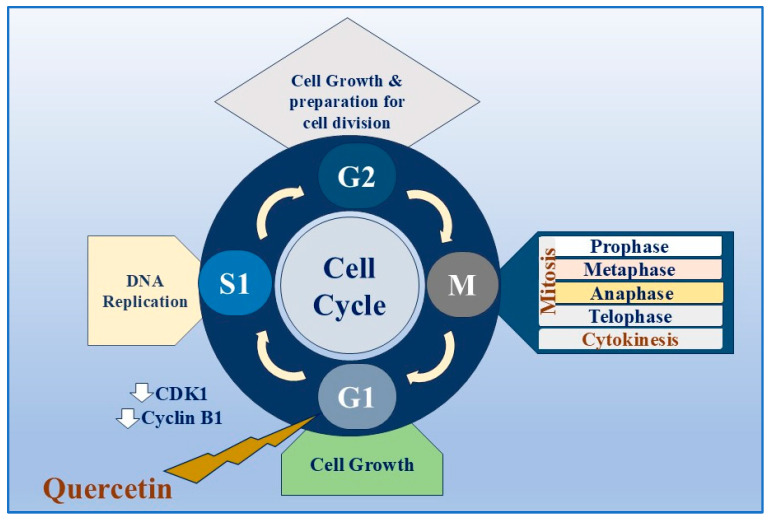
Quercetin induces cell cycle arrest in the G1 phase.

In vitro studies have demonstrated quercetin’s antitumor effect against melanoma [60] and pancreatic [61], breast [62], liver [63,64], and prostate [65,66] cancers. Quercetin’s anticancer effect is associated with its ability to control certain enzymatic processes, oxidative stress, and cellular pathways. When applied to tumours with multidrug resistance, quercetin has shown synergistic effects by suppressing the ejection of drugs facilitated by transporter proteins [54]. Furthermore, quercetin has the potential to induce autophagy and is effective in treating breast cancer by inhibiting the Akt-mTOR pathway in glycolysis and cell motility. Moreover, quercetin inhibited the growth and metastasis of breast cancer in mice with MCF-7 tumours. Additionally, it decreased the expression levels of VEGF, p-AKT, and PKM2 in the tumour tissues [67]. Quercetin’s antiproliferative effect can be primarily attributed to cell cycle arrest at the G1 phase, which occurs due to the downregulation of cyclin-dependent kinase 1 (CDK1) and cyclin B1 (Figure 3), which are essential components for the progression of the G2/M cell cycle, and the activation of phosphorylation for the retinoblastoma tumour suppressor protein, pRb [68]. In addition, the tumour-suppressing molecules Bax, p21Cip1, p27Kip1, cyt-c, caspase 3, caspase 8, and p53 are upregulated in prostate cancer after treatment with quercetin. Moreover, quercetin inhibited IL-6 and IL-10 cytokine production, resulting in the cytotoxicity of primary effusion lymphoma (PEL). In addition, it downregulated cell survival proteins, such as c-FLIP, cyclin D1, and cMyc, in PEL cells by inhibiting the PI3K/AKT/mTOR and STAT3 pathways [69] (Table 3).

When combined with other drugs, quercetin can increase the apoptotic effect of midkine, elevate caspase 3, and decrease the expression of the survivin gene. It decreased the number of S phase cells and induced G1-phase cell cycle arrest. Furthermore, quercetin increased the expression of PTEN while downregulating the phosphorylation of PI3K, Akt, ERK1/2, p38, NF-κB, and survivin proteins [65]. Using the human gastric cancer cell line AGS, Lei et al. investigated the potential therapeutic benefits of quercetin in combination with irinotecan/SN-38. Quercetin combined with SN-38 synergistically increased apoptosis and anti-proliferation, with alterations in GSK3β/β-catenin signalling. Treatment with quercetin either alone (twice weekly) or combined with irinotecan (10 mg/kg once weekly) resulted in significant inhibition of tumour growth, with lowered levels of COX-2 gene, and downregulation of tumour VEGF-R and VEGF-A [70].

One advantage of quercetin is its low toxicity; however, its shortcomings are low solubility and poor bioavailability, suggesting that nanoparticle encapsulation might improve its efficacy. Polymeric nanoparticles, stimuli-responsive polymeric nanoparticles, and non-responsive polymeric nanoparticles are a few examples. Additionally, quercetin-containing inorganic nanoparticles, including metal oxides, silica, and gold nanoparticles, have been studied [71].

##### Epigallocatechin-3-Gallate (EGCG)

One of the phenolic components of green tea (*Camellia sinensis*) is epigallocatechin-3-gallate (EGCG) [72]. It is also present in a wide variety of food herbs and plants, including strawberries, blackberries, cranberries, cherries, kiwis, pears, avocados, peaches, apples, pecans, pistachios, and hazelnuts [17]. EGCG, an ester of epigallocatechin and gallic acid, exhibits different biological and pharmacological actions, such as pro-apoptotic, anti-inflammatory, antiangiogenic, antioxidant, and antimetastatic properties [73], and has been used in clinical trials [74]. In addition, EGCG has been reported to have protective potential against neurodegenerative disorders, such as Parkinson’s and Alzheimer’s diseases [72].

Numerous health benefits, such as reduced circulating cholesterol, weight loss, cardiovascular protection, and inflammatory attenuation, have been demonstrated in different studies [75]. In addition, EGCG’s inhibitory effects on the initiation, development, and progression of several tumour types have been shown in several in vitro research using various cancer cell lines, as well as in vivo studies [76,77,78] (Table 3). Moreover, EGCG has been reported to decrease the side effects linked to chemotherapy and improve the therapeutic efficiency of existing treatments. EGCG has the potential to be a versatile anticancer drug by preventing cell cycle progression, triggering apoptosis, preventing invasion and metastasis, and modifying the tumour microenvironment (TME) [79]. Studies conducted on animals and cells have confirmed these effects and suggested several mechanisms through which EGCG acts as an anticancer agent. The mechanism by which EGCG functions as a potent antioxidant is mediated by reactive oxygen species (ROS). EGCG may also function as a pro-oxidant under specific conditions [80,81]. Despite its natural origin, safety, and affordability, its limited bioavailability is a significant challenge that is being addressed by encapsulating it in nanoparticles for further delivery [79].

In human hepatocellular liver cancer cells (HepG2), EGCG functions as a strong antioxidant that reduces oxidative stress by preventing the generation of ROS and increasing the activity of the antioxidant enzymes glutathione peroxidase and superoxide dismutase. [82]. Moreover, numerous studies have shown that EGCG downregulates MMP1 expression [83]. Considering that MMP1 plays a role in the migration, invasion, and metastasis of cancer cells [84], the anticancer effect of EGCG may be partly attributed to its suppression of MMP1 expression [81]. Furthermore, EGCG’s downregulation of MMP2 was suggested to decrease the phosphorylation of PI3K and ERK [81]. Moreover, EGCG inhibits cell division, migration, and Matrigel invasion in TW01 and NA nasopharyngeal cancer cells [85].

In a human pancreatic cancer xenograft model using AsPC-1 cells, EGCG suppressed human umbilical vein endothelial cell (HUVECs’) migration, capillary tube formation, and cell proliferation, and these suppressive effects were amplified in the presence of an ERK inhibitor. In addition, EGCG-treated mice tumour samples showed increased p38 and JNK activity and decreased ERK activity. EGCG and catechin gallate inhibited the induction of ConA-mediated MT1-MMP in U87 glioblastoma cells, while EGCG and gallocatechin gallate inhibited the induction of the endoplasmic reticulum stress (ERS) biomarker GRP78 and proMMP2 activation [86].

In cervical cancer HeLa cells, EGCG inhibited proliferation, induced apoptosis, and suppressed cell invasion and migration. Additionally, EGCG decreased MMP9 gene expression while upregulating TIMP1 gene expression [87]. Furthermore, EGCG induced apoptosis in HCT116 cells with wild-type p53 and HT-29 cells with mutant p53 in a manner independent of p53. Regardless of p53 status, EGCG inhibited MMP9 and VEGF expression [88]. HTLV-1-infected cells express Tax oncogenes. In ATL HuT-102 and C91-PL cells that were HTLV-1 positive, EGCG caused cytotoxicity and decreased Tax expression. In these cells, EGCG reduced MMP9 activity, NF-κB activity, and MMP9 mRNA and protein levels [89].

In bladder cancer SW780 cells, EGCG prevented the invasion, migration, and proliferation of cancer cells. Additionally, by activating caspases-8, -9, and -3, Bax, and poly-ADP ribose polymerase, EGCG induced apoptosis in these cells. When EGCG was injected into mice with SW780 tumours, both tumour weight and volume decreased. In SW780 tumours and cells, EGCG suppressed the expression of MMP9 and NF-κB at the protein and mRNA levels. The inhibitory effects of EGCG on the migration and proliferation of SW780 cells were cancelled by the addition of the NF-κB inhibitor SC75741 [90]. According to research about the effects of oral EGCG administration on patients with breast cancer receiving radiation therapy, EGCG decreased the activation of MMP2 and MMP9 in patient sera and decreased serum levels of VEGF and hepatocyte growth factor (HGF) in comparison to patients who were not receiving treatment [91].

Regarding clinical trials, oral delivery of EGCG was safe, practical, and efficient in a phase I clinical trial in unresectable stage III lung cancer when EGCG was combined with standard chemoradiotherapy, with a suggested dose of 440 µmol/L in a phase II clinical trial [92]. Despite the crucial significance of EGCG in cancer prevention, as demonstrated in different phase I and II clinical trials, additional trials are still required to fully comprehend the efficacy of EGCG in cancer treatment [11].

**Table 3:** The anticancer properties of flavonols in different types of cancer.

Flavonols	Anticancer Effect	Cancer Type	Citation
Quercetin	Increase the expression of PTEN while downregulation of the phosphorylation of PI3K, Akt, ERK1/2, p38, NF-κB, and survivin proteins.	Prostate cancer	[65]
	Induction of autophagy, inhibition of the Akt-mTOR pathway’s role in glycolysis and cell motility and reduction of the expression levels of VEGF, p-AKT, and PKM2 in tumour tissue.	Breast cancer	[67]
	Cell cycle arrest at the G1 phase that occurs due to the downregulation of cyclin-dependent kinase 1 (CDK1) and cyclin B1and Upregulation of Bax, Bcl-2, p21Cip1, p27Kip1, cyt-c, caspase 3, caspase 8, and p53.	Prostate cancer	[68]
	Inhibition of IL-6 and IL-10 cytokine production, resulting in cytotoxicity, in addition to downregulation of cell survival proteins, such as c-FLIP, cyclin D1, and cMyc via inhibiting the PI3K/AKT/mTOR and STAT3 pathways.	Primary Effusion Lymphoma	[69]
	Induction of apoptosis and anti-proliferation, with alteration of GSK3β/β-catenin signalling, in addition to reduction of COX-2 level, and downregulation of tumours’ VEGF-R and VEGF-A.	Human gastric cancer cell line AGS	[70]
Epigallocatechin-3-Gallate (EGCG)	Decreased cell adhesion and downregulated the expression of VEGF, NF-κB, FAK, and MT1-MMP.	Breast cancer MCF-7 cells	[93]
	Inhibition of cell division, migration, and Matrigel invasion.	TW01 and NA nasopharyngeal cancer cells	[85]
	Suppression of human umbilical vein endothelial cells (HUVECs’) migration, capillary tube formation, and cell proliferation and downregulation of MMP2, MMP7, MMP9, and MMP12 and reduction of the volume, angiogenesis, and metastasis of tumour, in addition to increased p38 and JNK activity and decreased ERK activity.	Pancreatic cancer xenograft AsPC-1	[94]
	Inhibition of cells proliferation, induction of apoptosis, suppression of cell invasion and migration, in addition to suppression of MMP9 gene expression while upregulation of TIMP1 gene expression.	Cervical cancer HeLa cells	[87]
	Inhibition of the levels of MMP9 and VEGF expression.	Colon cancer HCT116 cells	[88]
	Induction of cytotoxicity, decrease Tax oncogene expression, and reduction of MMP9 activity, NF-κB activity, and MMP9 mRNA and protein levels.	ATL HuT-102 and C91-PL cells	[89]
	Prevention of cell invasion, migration, and proliferation, in addition to activation of caspases-8, -9, and -3, Bax and poly-ADP ribose.	Bladder cancer SW780 cells	[90]
	Reduction of the activation of MMP2 and MMP9 in patient sera in addition to lowering the serum levels of VEGF and hepatocyte growth factor (HGF).	Patients with breast cancer	[91]

#### 5.1.4. Flavones: Luteolin

Luteolin (3,4,5,7-tetrahydroxy flavone), a naturally occurring flavone, is found in fruits and vegetables like celery, sweet bell peppers, chrysanthemum flowers, carrots, onion leaves, parsley, and broccoli [94]. It acts as an antioxidant or pro-oxidant biochemically and has a variety of biological effects, including anti-inflammatory, anti-allergic, and anticancer properties. It is an essential chemopreventive agent for the treatment of different cancers [95]. It can inhibit the proliferation of different types of tumour cells in vitro, with an IC_50_ ranging from roughly 3 to 50 μM [94]. Through a variety of mechanisms, such as kinase suppression, cell cycle regulation, induction of apoptosis, and reduction of transcription factors, luteolin has been shown to impede the progression of carcinogenesis, including cell transformation, metastasis, invasion, and angiogenesis (Table 4). The anticancer properties of luteolin also include DNA damage, redox regulation, and inhibition of cancer cell proliferation, which are linked to apoptosis induction [95].

Luteolin demonstrated cell cycle arrest during the G1 phase in a variety of human cancer cell lines, including gastric, prostate, and melanoma. The induced G1 cell cycle arrest was associated with the inhibition of CDK2 activity in colorectal cancer HT-29 and melanoma OCM-1 cells. In MCF-7 breast cancer cells induced by EGF, luteolin showed significant suppression of the expression of p-STAT3, p-EGFR, p-Akt, and p-Erk1/2 and inhibited cell proliferation. Additionally, it was able to inhibit the EGFR signalling pathway in human breast cancer cell lines. Moreover, previous studies have shown that a moderate dose of luteolin (10 mg/kg) can prevent the establishment of large tumours in a 7,12-dimethylbenz (a)anthracene-induced tumour model and can dramatically reduce the levels of vascular endothelial growth factor (VEGF) in Sprague-Dawley rats. In addition, luteolin suppressed the growth of MPA-dependent human breast cancer cell xenograft tumours, progestin-dependent VEGF release from breast cancer cells, and tumour cell survival. Furthermore, it reduced blood vessel density and prevented T47-D and BT-474 breast cancer cells from acquiring stem cell-like characteristics [94].

Luteolin inhibited progression of MCF-7 breast cancer cells in the G1 phase and induced sub-G1 cell population, altered the morphology of the nucleus, raised the mRNA levels of death receptors such as DR5 and caspase and inhibited poly-ADP ribose polymerase, a key indicator that helps a cancer cell heal itself in a dose-dependent manner, and increased caspase-9/-8/-3 activity. Furthermore, luteolin induced the release of cytochrome c after impairing the potential of the mitochondrial membrane. As a result, Bcl-2 expression was suppressed, and Bax expression increased [96]. Similarly, by inducing apoptosis, regulating the cell cycle, and inhibiting proliferation, luteolin exhibited anticancer activity against MDA-MB-543 cells. In MDA-MB-231 cells, luteolin caused cell cycle arrest in the S phase by lowering telomerase levels and preventing the phosphorylation of NF-κB inhibitor α. As a result, it lowered the mRNA levels of human telomerase reverse transcriptase, which encodes the catalytic portion of telomerase. Additionally, luteolin inhibits the growth of malignant breast cells and triggers apoptosis, leading to the inhibition of cancer spread [97]. The synergistic effects of luteolin and celecoxib treatment were observed in MCF-7 and MCF7/HER18 cells through Akt inactivation and extracellular signal-regulated kinase (ERK) signalling inhibition [98].

It has been shown that luteolin mono-acylated derivatives exhibit anticancer and antioxidant properties against HCT116 colon cancer cells. The resulting compounds become more lipophilic upon acylation of the -OH groups, increasing their bioavailability [99]. Luteolin inhibited the G2/M phase of the cell cycle and caused colon cancer cells to undergo apoptosis. In a dose- and time-dependent manner, luteolin had an inhibitory effect on cell proliferation in LoVo human colon cells by triggering cell cycle arrest at the G2/M phase and inhibition of cyclin B1/CDC2. Apoptotic protease-activating factor 1 (APAF1) is stimulated by deoxyadenosine triphosphate, which controls these processes [100]. You and colleagues documented how luteolin inhibits colon tumours through apoptosis and autophagy. After treatment with luteolin, HCT116 cells displayed increased p53 phosphorylation and p53 target gene expression, which leads to cell cycle arrest and apoptosis. Thus, luteolin-induced p53 wild-type cells to undergo autophagy. This suggests that autophagy induced by the compound depends on p53 [101].

Through the increased Nrf2 transcription induced by DNA demethylation of its promoter, luteolin exerts anticancer effects on colorectal cancer cells. Furthermore, by strengthening the interaction between Nrf2 and p53, luteolin increases the expression of antioxidant enzymes and apoptotic proteins [102]. Additionally, Lutein has been demonstrated to inhibit the proliferation of colorectal malignant cells, interrupt the cell cycle, damage DNA, and accelerate apoptosis by targeting the MAPK pathway. These findings suggest that luteolin may be a useful adjuvant for the treatment of colorectal cancer in the future [103].

Luteolin has been reported to exert its pharmacological effects by inhibiting the expression of cyclin E, MMP-2, cyclin D1, vimentin, Bcl2, and N-cadherin while promoting the expression of E-cadherin, Bax, and p21. In gastric tumour cells, luteolin’s anticancer properties are confirmed by a decrease in the expression of p-PI3K, p-mTOR, p-AKT, p-STAT3, and Notch1, and an increase in the amount of p-P38 signal transduction [104]. Accordingly, luteolin (40 mg/kg) effectively inhibited the growth of cancer in BGC-823 gastric tumour xenografts in experimental mice. According to published studies, luteolin inhibits the activation of the immune system and the expression of MMP-9 and VEGF-A, which prevents cancer growth. Furthermore, in a c-Met-overexpressing individual-derived xenograft model, luteolin significantly suppressed the growth of cancer and decreased the expression of c-Met, ki-67, and MMP-9 in malignant tissues. Moreover, luteolin promoted apoptosis and suppressed invasiveness and proliferation in gastric tumour cells that overexpressed c-Met (SGC7901 and MKN45). Additionally, it downregulated MMP-9 and enhanced the activation of apoptosis-related proteins, such as multi (ADP-ribose) polymerase-1 and caspase-3. Furthermore, luteolin decreased c-Met expression and phosphorylation while knocking down ERK and Akt phosphorylation. It was also found that c-Met was not necessary for the downstream phosphorylated levels of Akt [105].

Luteolin-induced apoptosis in vitro suppressed the growth of tumour cells in vivo by significantly inhibiting the invasion, migration, and proliferation of stomach tumour cells in a time- and dose-dependent manner. In this regard, luteolin therapy caused EMT reversion by shrinking the cytoskeleton and increasing the expression of E-cadherin downstream of mesenchymal markers, such as vimentin, N-cadherin, and Snail. Additionally, it prevents the transduction of Notch1 signals [106]. Moreover, Lutein treatment of GC cells reduced the expression of the target genes Mcl-1 and Bcl-xl and survival while also inhibiting STAT3 phosphorylation. Furthermore, in vivo, research validated luteolin’s inhibitory effects on tumour growth and progression [107].

In lung malignant cells, luteolin promotes the production of ROS, which in turn mediates the expression of the tumour necrosis factor-activated cascade. By upregulating c-Jun N-terminal kinase expression and downregulating NF-κB expression, luteolin promoted tumour necrosis factor-induced apoptosis in lung cancer cells. Luteolin also targets a variety of cancer pathways, such as redox stress, ROS formation, cell cycle arrest, autophagy induction, apoptosis initiation, and suppression of cell proliferation, all of which lead to the death of tumour cells [108].

Cai and colleagues suggested that luteolin inhibits the cell cycle and promotes apoptosis by increasing the synthesis of Bax, JNK activation, and enhancing the cleavage of caspase-3 and procaspase-9 in lung cancer cells (A549). Additionally, it inhibits trans-nuclear translocation controlled by TNF-α and NF-kB [109]. Luteolin inhibited cell growth and triggered apoptosis by increasing caspase-9 and -3 activation, decreasing Bcl-2, increasing Bax expression, phosphorylating MEK and its downstream kinase ERK, and activating Akt. Moreover, suppression of MEK-ERK signalling suggests that the pro-apoptotic and anti-migration effects of luteolin are significantly mediated by the MEK-ERK signalling pathway [110].

Through the regulation of both intrinsic and extrinsic cascades, which were suppressed by z-Val-Ala-Asp fluoromethyl ketone, luteolin-induced apoptosis in NCI–H460 human non-small cell lung cancer cells. This suggests that luteolin promotes caspase-dependent apoptosis. Additionally, luteolin-induced autophagy has been discovered to be a mechanism of cell death [111]. Another study showed that luteolin has anticancer effects by increasing Sirt1-regulated apoptotic cell death in NCI-H460 cells [112,113]. Moreover, it increased cleaved caspase-3 levels and reduced cyclin D1 expression by decreasing the mRNA levels of LIM domain kinase signalling-related targets, such as p-cofilin and phosphorylated LIM domain kinase. Furthermore, luteolin reduced phosphorylated LIM domain kinase, Ki-67, and p-cofilin levels, all of which inhibited the development of tumours in a xenograft model of lung tumour patients [113].

Macrophages linked to tumours are essential for the development of cancer [114]. According to Choi et al., luteolin lowers the mRNA levels of M2-associated genes and prevents the attachment of a phosphate group to STAT6, a significant IL-4 downstream signal. Additionally, they found that luteolin inhibited the migration of Lewis lung cancer cell lines in a manner dependent on chemokine (C–C motif) ligand 2 [115].

Ionising radiation and luteolin combination therapy increased programmed cell death in lung cancer cells by downregulating Bcl-2, which in turn stimulated caspase-9, -8, and -3. Additionally, luteolin led to the accumulation of ROS and the addition of phosphate to p38 MAPK. Moreover, in the NCI–H460 cell xenograft mouse model, combined therapy with luteolin and ionising radiation increased programmed cell death and suppressed the progression of cancer. This substance may act as a radiosensitiser, promoting programmed cell death by activating the p38/ROS/cascade pathway [116].

In contrast, luteolin has been reported to significantly reduce the proliferation of oral cancer stem cell lines and the activities of acetaldehyde dehydrogenase and CD44-positive cells. It has also been suggested that luteolin reverses the radiosensitivity of oral tumour cells, as the combined therapy of luteolin and radiation significantly decreases the invasion and spread of oral cancer [117]. Moreover, luteolin demonstrated cytotoxicity against human immortalised keratinocytes (HaCaT) and human melanoma (A375) cells in skin cancer. Furthermore, HaCaT cell lines accumulated cells in the G2/M phase, and A375 cell lines accumulated cells in the G0/G1 phase when luteolin is incubated with cancer cell lines [118].

By reducing miR-301-3p, luteolin decreased the proliferation of pancreatic ductal adenocarcinoma (PDAC) cells and enhanced the antiproliferative effect of TRAIL on tumour cells [119]. In female Syrian golden hamsters, luteolin (100 ppm) reduced carcinogenesis by increasing amylase activity and reducing PDAC incidence and multiplicity, Ki-67 labelling index, pSTAT3 signal transduction, and neoplastic lesion development [120]. Additionally, luteolin (150 and 75 mg/kg) prevented tumour growth in xenografted SCID mice [121].

By inducing apoptosis, decreasing extracellular matrix contraction, and inhibiting growth, luteolin exerts chemopreventive therapeutic effects against prostate cancer. MDM2 was suppressed by luteolin, and luteolin-induced E-cadherin expression decreased due to active Akt overexpression. Therefore, in prostate cancer, luteolin affects E-cadherin expression through the Akt/MDM2 pathway. Furthermore, by suppressing the expression of androgen receptors, luteolin reduced the expression of prostate-specific antigens. It reduced the mRNA levels of numerous genes involved in the cell cycle and epidermal growth factor receptor signal transduction cascades. Luteolin significantly promoted cell cycle arrest at the G2/M phase and triggered the production of p21 RNA and c-FOS. Different studies have revealed that c-FOS or p21 silencing RNAs significantly reduce the expression of RNA of their respective targets, but they have no effect on cell proliferation, and neither double silencing RNA nor single silencing RNA can stop the proliferation of prostate cancer cells [94].

According to Cao et al., luteolin decreased the viability of SMMC-7721 liver cancer cells in a manner that is dependent on both time and dose. Moreover, luteolin decreased Bcl-2 expression at the mRNA and protein levels, increased caspase 8 expression, and caused G0-/G1-phase arrest. Lastly, co-administration of the autophagy inhibitor chloroquine reduced the impact of luteolin on cell death [122].

Nazim and Park [123] showed how luteolin and TRAIL therapy work together, as well as how they affect TRAIL-resistant Huh7 cells. The synergetic effect of luteolin and VV-IL-24 (oncolytic vaccinia virus) to decrease tumour growth through single therapy was validated by Wang et al. [124]. They reported that luteolin inhibited the activation of the PI3K/Akt and NF-κB signalling pathways, which are implicated in the growth and survival of cancer cells. Additionally, it increased the cytotoxicity of chemotherapeutic drugs in kidney cancer cells, indicating that it may be used along with conventional medications to boost their effects. Moreover, luteolin reduces the negative effects of chemotherapy, which makes it a desirable drug for the treatment of kidney cancer.

Nanotechnology is a novel chemoprevention technique for delivering luteolin. To explore its anticancer capabilities against head, neck, and lung cancers, hydrophobic luteolin was synthesised to produce water-soluble polymer-encapsulated nano-luteolin. Nano-luteolin, like luteolin, has been demonstrated to inhibit the growth of lung cancer cells (H292 cell line) and squamous cell carcinoma of the head and neck (SCCHN) cells (Tu212 cell line) in vitro. Using a tumour xenograft mouse model, in vivo experiments comparing nano-luteolin to luteolin revealed that the latter greatly suppressed the growth of SCCHN cancer. This suggests that luteolin may be used for chemoprevention in clinical settings [125].

**Table 4:** The anticancer properties of luteolin in different tumour cells.

Anticancer Effect of Luteolin	Cancer Type	Citation
Inhibition of CDK2 activity and induction of induced G1 cell cycle arrest.	Colorectal cancer HT-29 and melanoma OCM-1 cells	[94]
Suppression of the expression of p-STAT3, p-EGFR, p-Akt, and p-Erk1/2, as well as inhibition of cell proliferation.	MCF-7 breast cancer cells	[94]
Increase of the mRNA levels of death receptors such as DR5 and caspase-9/-8/-3 activity, in addition to inhibition of poly-ADP ribose polymerase. Moreover, the induction of the release of cytochrome c after impairing the potential of the mitochondrial membrane. As a result, Bcl-2 expression was suppressed, and Bax expression rose.	MCF-7 breast cancer cells	[96]
Induction of cell cycle arrest in the S phase by lowering telomerase levels and preventing the phosphorylation of NF-κB inhibitor α, in addition to inhibition of the growth of breast malignant cells and induction of apoptosis.	MDA-MB-231 breast cancer cells	[97]
Akt inactivation and extracellular signal-regulated kinase (ERK) signalling inhibition.	MCF7/HER18 breast cancer cells	[98]
Induction of cell cycle arrest at the G2/M phase and inhibition of cyclin B1/CDC2.	LoVo human colon cells	[100]
Increased p53 phosphorylation and p53 target gene expression, which leads to cell cycle arrest, apoptosis, and autophagy.	HCT116 colon cells	[101]
Increased Nrf2 transcription by the DNA demethylation of its promoter, in addition to strengthening the interaction between Nrf2 and p53 that results in increased expression of antioxidant enzymes and apoptotic proteins.	Colorectal cancer cells	[102]
Inhibition of the proliferation of colorectal malignant cells, interruption of the cell cycle, damaged DNA and accelerated apoptosis through targeting the MAPK pathway.	Colorectal cancer cells	[103]
Decrease in the expression of p-PI3K, p-mTOR, p-AKT, p-STAT3, and Notch1, and an increase in the amount of p-P38 signal transduction.	Gastric tumour cells	[104]
Inhibition of the immune system and the expression of MMP-9 and VEGF-A, which stops cancer from growing, in addition to suppression of the expression of c-Met, ki-67, and MMP-9 that results in inhibition of tumour cells invasiveness and proliferation and induction of apoptosis.	Gastric tumour cells	[105]
EMT reversion by shrinking the cytoskeleton and increasing the expression of E-cadherin downstream of mesenchymal markers such as vimentin, N-cadherin, and Snail, in addition to prevention of the transduction of Notch1 signals.	Gastric tumour cells	[106]
Reduction of the expression of the target genes Mcl-1 and Bcl-xl and survival, in addition to inhibition of STAT3 phosphorylation.	Gastric cancer cells	[107]
Induction of ROS production, which in turn mediates the expression of tumour necrosis factor-activated cascade, in addition to upregulation of c-Jun N-terminal kinase expression and downregulation of NF-κB expression that results in promotion of tumour necrosis factor-induced apoptosis.	Lung cancer cells	[108]
Increase the synthesis of Bax, activation of JNK, and enhancing the cleavage of caspase-3 and procaspase-9, in addition to inhibition of trans-nuclear translocation controlled by TNF-α and NF-kB.	Lung cancer cells (A549)	[109]
Activation of caspase-9 and -3, inhibition of Bcl-2, increasing Bax expressions, phosphorylation of MEK and its downstream kinase ERK, activation of Akt, inhibition of cell growth, and induction of apoptosis.	Lung cancer cells (A549)	[110]
Increased cleaved caspase-3 levels and reduced cyclin D1 expression by decreasing the mRNA levels of LIM domain kinase signalling-related targets, such as p-cofilin and phosphorylated LIM domain kinase, that results into inhibition of tumour development.	Lung tumour xenograft	[111]
Reduction of miR-301-3p level results in inhibition of tumour cells proliferation and enhancing the antiproliferative effect of TRAIL on tumour cells.	Pancreatic ductal adenocarcinoma cells (PDAC)	[119]
Suppression of the expression of androgen receptors and prostate-specific antigen, in addition to the reduction of the mRNA levels of numerous genes involved in the cell cycle, cascades and the epidermal growth factor receptor signal transduction cascade that significantly promoted cell cycle arrest at the G2/M phase and triggered the production of p21 RNA and c-FOS.	Prostate cancer cells	[94]
Decreased Bcl-2 at the mRNA and protein levels, increased caspase 8, and caused G0-/G1-phase arrest. Additionally, it increased Beclin 1 expression, expedited the conversion of LC3B–I to LC3B–II, and increased the number of intracellular autophagosomes.	SMMC-7721 liver cancer cells	[122]
Inhibition of the activation of the PI3K/Akt and NF-κB signalling pathways, which are implicated in the growth and survival of cancer cells.	Kidney malignant cells	[124]

#### 5.1.5. Flavanones

The 40-methoxy derivative of the flavanone eriodictyol is called hesperetin (HSP), and its IUPAC name is 5,7-dihydroxy-2-(3-hydroxy-4 methoxyphenyl)-2,3-dihydrochromen-4-one [126]. HSP, a naturally occurring flavonoid with a variety of pharmacological characteristics, is mostly present in citrus fruits such as Citrus aurantinum, Citrus sinensis, and Citrus limon [127].

HSP may be a promising cancer treatment candidate (Table 5) because it demonstrates a cytotoxic mechanism against a variety of cancer cells, including breast [128], pancreatic [129], prostate [130], glioblastoma [131], liver [132], kidney [133], colon [134], lung [135], oral [136], [137], osteosarcoma [138], ovarian [139], thyroid [140], leukaemia [141], and other cancers [126]. HSP has been shown in numerous studies to be a promising treatment for breast cancer. HSP may promote DNA damage and apoptosis while inhibiting the growth, viability, migration, invasion, mammosphere formation, and colony formation of cancer cells. In MCF-7 breast cancer cells, it increased the mRNA levels of p53, NOTCH1, and PPARG and decreased β-catenin, leading to apoptosis and cell cycle arrest in the G0/G1 phase [142].

In breast cancer, tumour suppressor genes that regulate cell cycle progression are upregulated by HSP. HSP induces both intrinsic and extrinsic pathways that lead to cell death. In addition, HSP can inhibit certain tumor-related growth factors, which will prevent metastasis, inhibit MMP-9 production, and arrest the cell cycle in the Sub G1 phase [126]. According to a recent study, when MCF-7 breast cancer cells were treated with HSP (1–20 μM), aryl hydrocarbon receptor (Ahr) was inhibited, and the expression of CYP1A1, 1A2, and 1B1 was downregulated [143]. Furthermore, HSP inhibited the activity of the aromatase enzyme, cyclin D1, CDK4, Bcl-xL, and pS2, and it increased the expression of CCAAT/C/EBP, pERK-1&-2, and p57Kip2. These actions helped decrease tumour growth in MCF-7 breast cancer cells and female athymic mouse models, both in vitro and in vivo [144]. At a concentration of 95 μM, HSP reduced HER2, MMP-9, and Rac1 expression, lamellipodia formation, and arrested the cell cycle at the G2/M phase, thereby lowering cell viability, invasion and migration, and promoting apoptosis, according to research conducted on HER2 overexpressed breast cancer cells (MCF-7/HER2) and MCF-7/EV cells [145]. In MCF-7, MCF-10A, HMEC, and MDA-MB 231 breast cancer cells, HSP (20–200μM) was found to increase ROS production, cyto-C release, Bax/Bcl-2 ratio, PARP cleavage, caspase-9, -3, -7, JNK, and sk1 activation, in addition to the activation of the ASK1/JNK pathway [146]. In MDA-MB-231 breast cancer cells, HSP suppressed insulin receptor-beta subunit (IR-beta) phosphorylation and Akt, which lowered glucose absorption, leading to decreased cell proliferation [147]. HSP reduced the growth of MDA-MB-231 breast cancer cells by inhibiting HER2-tyrosine Kinase (HER2-TK) activity, causing MMP loss, chromatin condensation, and activating of caspase-8 and-3 [148]. This resulted in cell cycle arrest in the G2 phase and lowered SKBR3. Furthermore, HSP was able to induce apoptosis and prevent metastasis in 4T1 murine breast cancer cells by downregulating MMP-9 production and stopping the cell cycle at the Sub G1 phase [149].

HSP has been known to play a significant role in reducing the risk of prostate cancer and successfully treating it [136]. G0/G1 phase arrest was observed after HSP treatment via increased phosphorylation of the signal transducer and activator of transcription 3 (STAT 3), extracellular signal-regulated kinase ½ (ERK1/2), and AKT signalling pathways, as well as IL-6 gene expression [150]. HSP is also linked to cell cycle arrest at the G1/S phase and elevated mitochondrial membrane depolarisation, leading to apoptosis and decreased cell viability [151].

In H522 lung cancer cells, HSP induced apoptosis by initiating the Fas death receptor/extrinsic pathway, which led to the dose-dependent upregulation of Bax, caspase-3, and caspase-9 [135]. Similarly, by blocking transforming growth factor β and decreasing glucose uptake in cancer cells by downregulating glucose transporter expression, HSP demonstrated strong antiproliferative effects in H441 lung cancer cells [152]. When used with copper, HSP was able to inhibit angiogenesis via the mitochondria-mediated pathway by activating the TRIAL cytotoxic protein, which triggers many mechanisms of apoptosis [153]. Additionally, HSP inhibits IL-1β, which reduces COX-2 expression and PGE2 generation in A549 lung cancer cells [154]. By reducing LPO and altering antioxidant enzymes such as NF-kB, PCNA, and CYP1A1, HSP was able to inhibit the development of cancer in Swiss albino mice. This investigation demonstrates that HSP’s free radical-scavenging, antioxidant, anti-inflammatory, and antiproliferative properties of HSPs have the potential to prevent B[a] P-induced lung cancer [155]. Through the activation of mitochondrial-mediated pathways, HSP suppresses cell viability and proliferation while enhancing apoptosis. In a HSP dose-dependent manner, Bcl2 was downregulated while ROS, ATP, Ca^2+^, Cyto-C, AIF, and Apaf-1 were increased [156].

In the human cancer cell line HCT-116, HSP therapy activates the c-Jun-N-Terminal kinase (JNK) pathway, which reduced cell viability and induced apoptosis [157]. By elevating Bax and Caspase 3 levels and concurrently downregulating the anti-apoptotic protein BCL-2, HSP demonstrated an inhibitory effect on human cancer cell HT-29 through the induction of mitochondrial-mediated apoptosis [158].

HSP has the potential to treat liver cancer by inducing apoptosis and damage to cancer cells and reducing liver injury, liver enlargement, and hepatic fibrosis [126]. Miler et al. [159] found that oral administration of HSP at a dose of 15 mg/kg enhanced the death of cancer cells in male Wistar rats. It also increased the activity of antioxidant enzymes like catalase (CAT), glutathione reductase (GR), and superoxide dismutase (SOD). In Sprague-Dawley rats, HSP (50 mg/kg/day) induced apoptosis and controlled oxidative stress by increasing the expression of Fas/FasL and caspase-8, -3, and albumin levels while decreasing the levels of hepatic glutathione (GSH), hepatic malondialdehyde (MDA), and Bcl-2 [132]. Furthermore, Kong et al. discovered that HSP reduced liver fibrosis and induced apoptosis in HSC-T6 cells and male C57 mice. It inhibited the TGF-β1/Smad pathway and reduced the levels of AST, ALT, hydroxyproline (Hyp), HA, LN, TNF-α, IL-6, extracellular matrix (ECM) production, and Smad2/3 phosphorylation. In Littermate male C57BL/6J mice, HSP derivative reduced the levels of ALP, ALT, AST, TGF-β1, HA, Hyp, F4/80þ macrophage infiltration, MCP-1, TNF-α, IL-1β, IL-6, TNF-α, and IL-1β, Gli-1, and Shh expression at concentrations ranging from 25 to 100 mg/kg [160]. Additionally, HSP induced apoptosis in LX-2 liver cells by decreasing the expression of α-SMA, Col1α1, Col3α1, TIMP-1, PAI-1, and Gli-1 and increasing the levels of Bax and Caspase-3 [161]. In addition, HSP decreases Bcl-2, mitochondrial AIF, mitochondrial Apaf-1, and mitochondrial cyt-c, which drive cancer cell apoptosis, while upregulating a few intracellular ROS, ATP, Ca^2+^, and cytosolic components such as AIF, Apaf-1, cyt-c, caspase-3, caspase-9, and Bax [156].

In recent years, HSP have shown promise in the treatment of pancreatic cancer [126]. In a study using Miapaca-2, Panc-1, and SNU-213 cell lines at different doses, Lee and his colleagues discovered that HSP (0–20 μM) inhibited the migration of the treated cells. Furthermore, HSP treatment at a dosage of 2.5 μM significantly decreased the viability of Panc-1 pancreatic cancer cells. Additionally, apoptosis was induced because HSP obstructed intracellular signalling, including focal adhesion kinase (FAK), p38 phosphorylation, and caspase-3 activation. Furthermore, HSP at 30 mg/kg exhibited anti-growth properties through the activation of caspase-3 in a Panc-1 xenograft model in BALB/c nude mice [129].

Additionally, HSP may be used to treat renal cancer [126]. HSP decreased oxidative stress, lipid peroxidation, MDA, TNF-α, IL-1β, and IL-6 levels, thereby reducing cisplatin-induced nephrotoxicity in rats [162]. Moreover, HSP reduced renal fibrosis, normalised renal function in the (NRK)-52E cell line and UUO-mouse model, and decreased the expression of fibronectin (FN), Collagen I, α-SMA, EMT, Shh, Gli-1, and E-cadherin [163]. Furthermore, HSP control signalling pathways, metastasis, and some inflammatory indicators, in addition to activating genes linked to antioxidant enzymes. Thus, HSP may exhibit anticancer activity in kidney cancer [126].

In vitro and in xenograft tumours, HSP inhibited the proliferation of gastric cancer cells by inducing apoptosis through a dose-dependent increase in the Bax/Bcl-2 ratio, cyt-c, caspase-3, caspase-9, AIF, and Apaf-1 via a mitochondrial-dependent mechanism [156]. Moreover, HSP reduces cell migration and invasion in gastric cancer cells by inhibiting the expression of genes linked to metastasis and lowering disruptor of telemetric silencing 1-like (DOT1L) and histone H3K79 methylation by controlling CBP activity [164]. In addition, HSP can elevate the production of ROS to induce apoptosis in SK-OV-3 ovarian cancer cells [139]. By changing the endoplasmic reticulum stress signalling pathway, hesperidin (the aglycone form of HSP) inhibits the growth of (A2780) ovarian cancer cells and triggers apoptosis [165]. HSP also demonstrated cytotoxicity against ovarian cancer cells. These phytochemicals activate cleaved caspase-3 in ovarian cancer cells, promoting antioxidant activity and inducing apoptosis [166].

In U251 and U87 glioblastoma cells, HSP reduced cell viability by decreasing Bcl-2 and increasing Bax protein expression, thus inducing apoptosis in a dose-dependent manner. Moreover, it also caused cell cycle arrest by decreasing cyclin B1 CDK1 and enhancing tumour suppressor gene p21 activation via p38 MAPK, which arrests the G2/M phase [167]. Moreover, HSP controls apoptosis and cell division by generating ROS and activating the SOD enzyme [168]. When C6 glioma cells were implanted in Wistar rats, HSP prevented tumour growth by activating caspase-3 and -9, raising the Bax/Bcl-2 ratio, which caused apoptosis, and downregulating the HIF-1α, VEGF, and VEGFR2 signalling pathways. It also decreased the expression of cyclin B1 and D1 while increasing the expression of Claudin-1 and ZO-1, which decreased the growth of cancer cells [169].

HSP exhibited an anticancer effect on leukaemia HL60 cell lines by inducing apoptosis via increasing caspase-3 activity, MMP loss, and cell cycle arrest in the G2/M and G0/G1 phases [141]. In K562 leukaemia cells, HSP induced apoptosis, arrested the G0/G1 phase, and increased the expression of the DUSP1 (dual specificity phosphatase 1), DUSP3, DUSP5, CDK1A, CDK1B, GADD45B, SPRR2D, MT1F, MT1A, p27Kip1, CASP4, and NFKBIA genes [170]. Furthermore, HSP increased the production of BAD, caspase-3, luciferase activity, PARP cleavage, and Notch 1 signalling [140]. Additionally, HSP increased ROS generation and JNK1/2, p38, Bax, and p21 expression in A431 human cancer cells while suppressing ERK1/2, cyclin B1, D1, D3, and E1 expression, which resulted in apoptosis and decreased cell viability [171].

By stimulating PI3K-Akt signalling, cytotoxic T lymphocytes, and the tolerogenic T cell response, HSP can suppress melanogenic tumour growth [172]. HSP caused oesophageal Eca-109 cancer cells to undergo apoptosis both in vitro (Eca-109) and in vivo (female BALB/c nude mice). Furthermore, the HSP-treated Eca-109 cell line showed decreased PI3K/AKT signalling pathway, cyclin D1, MMP-2,9, and PI3K-p85 expression, as well as increased PTEN phosphorylation and p21 expression, which results in cell cycle arrest at the G0/G1 phase [173].

Hyperplasia, dysplasia, and increased cell proliferation in squamous cell carcinoma (SCC) are abnormalities induced by DMBA-induced oral tumour development in the buccal pouches. HSP treatment has been linked to anticancer effects by mediating apoptotic and antiproliferative effects. HSP inhibits cell proliferation in the buccal mucosa of DMBA-treated animals by downregulating vascular endothelial growth factor (VGEF) in DMBA-treated tissue [174].

It was reported that Hesperetin and Naringenin (Nar), two flavanones, target the mitochondrial fission pathway to exert anticancer effects. They correct abnormal mitochondrial dynamics and lipid metabolism by targeting Drp1, which causes ER stress and apoptosis [175]. The combination of these natural compounds decreased the adverse effects of several medications and showed great benefits against cancer [126]. Phosphorylation of FAK and the p38 signalling pathway were downregulated when HSP was administered in combination with naringin and naringenin, although this was not the case with either of the two treatments [176]. In a xenograft model, Wang et al. found that co-treatment of HSP with platinum caused apoptosis-related cell death by downregulating UGT1A3 and concurrently increasing ROS [177]. Moreover, HSP and luteolin together enhanced the death of MCF-7 breast cancer cells [178]. Dextran and HSP combination enhanced the HSP’s antioxidant activity and had a greater cytotoxic effect on MCF and AGS than when HSP was used alone [179]. Additionally, the combination of HSP and doxorubicin arrested the cell cycle in the G2/M phase and prevented metastasis by suppressing the production of MMP-9 in 4T1 cells [149]. Moreover, HSP and 5-FU (fluorouracil) together suppressed cell proliferation in oesophageal cancer Eca-109 cells by downregulating Bcl-2 and increasing cleaved caspase-3 and caspase-9 more efficiently than either drug alone [173].

HSP can also be used as an adjuvant treatment for multidrug resistance. Excessive doxorubicin use results in drug resistance by increasing drug efflux and overexpressing P-glycoprotein (P-gp). However, by reducing the optimum concentrations of both HSP and doxorubicin, HSP combined with doxorubicin therapy inhibits P-gp expression in MCF-7 and MCF-7/DOX cells [180]. Additionally, certain anticancer medications increase the sensitivity of resistant cell lines when NF-κB and IGF1R expressions are inhibited [181]. When HSP was administered to A549/DDP cells, P-gp-mediated MDR was reversed by lowering P-gp expression, which was directly associated with the suppression of the transcription factor NF-κB signalling pathway [182].

Interestingly, Eudragit-E nanoparticles loaded with HSP (HETNPs) showed anticancer efficacy in oral carcinoma (KB) cells. HETNPs more successfully demonstrate elevated ROS levels, loss of mitochondrial membrane potential, and apoptotic morphological alterations than native HSP [183]. Moreover, collagen, nicotinamide adenine dinucleotide (NAD), and flavin adenine dinucleotide (FAD) emissions were reduced in oral carcinoma caused by 7,12-Dimethylbenz[a]anthracene (DMBA); however, oral administration of HSP and its nanoparticles restored the endogenous fluorophore emission and increased the redox ratio in the buccal mucosa of DMBA animals [184].

**Table 5:** Anticancer effects of hesperetin in different tumour cells.

Anticancer Effect of Hesperetin (HSP)	Cancer Type	Citation
Increased the mRNA levels of p53, NOTCH1, and PPARG and decreased β-catenin, leading to apoptosis and cell cycle arrest in the G0/G1 phase.	MCF-7 breast cancer cells	[142]
Upregulation of tumour suppressor genes that can regulate cell cycle progression, induction of both intrinsic and extrinsic pathways that lead to cell death, inhibition of certain tumour-related growth factors which will prevent metastases, inhibition of MMP-9 production and induction of cell cycle arrest in the Sub G1 phase.	Breast cancer cells	[126]
Inhibition of the aryl hydrocarbon receptor (Ahr) and downregulation of the expression of CYP1A1, 1A2, and 1B1.	MCF-7 breast cancer cells	[143]
Inhibition of the activity of the aromatase enzyme, cyclin D1, CDK4, Bcl-xL, and pS2, while increasing the expression of CCAAT/C/EBP, pERK-1&-2, and p57Kip2 that results in decrease the tumour growth.	MCF-7 breast cancer cells and female athymic mice models	[144]
Reduction of HER2, MMP-9, Rac1 expression, lamellipodia formation, and induction of cell cycle at the G2/M phase, thereby lowering cell viability, invasion, migration, and promoting apoptosis.	HER2 overexpressed breast cancer cells (MCF-7/HER2) and MCF-7/EV cells	[145]
Increase ROS production, cyto-C release, the Bax/Bcl-2 ratio, PARP cleavage, caspase-9, -3, -7, JNK, and activation of sk1 and the ASK1/JNK pathway.	MCF-7, MCF-10A, HMEC, and MDA-MB 231 breast cancer cells	[146]
Suppression of insulin receptor-beta subunit (IR-beta) phosphorylation and Akt, which lowers glucose absorption, leading to decreased cell proliferation.	MDA-MB-231 breast cancer cells	[147]
Inhibition of HER2 Tyrosine Kinase (HER2-TK) activity, leading to MMP loss, chromatin condensation, and activating caspase-8 and-3 that resulted in cell cycle arrest at the G2 phase and lowered SKBR3.	MDA-MB-231 breast cancer cells	[148]
Induction of apoptosis and prevention of metastasis of tumour cells by downregulating MMP-9 production and stopping the cell cycle at the Sub G1 phase.	4T1 murine breast cancer cells	[149]
G0/G1 phase arrest via increasing phosphorylation of the signal transducer and activator of transcription 3 (STAT 3), extracellular signal-regulated kinase ½ (ERK1/2), and AKT signalling pathways, as well as IL-6 gene expression.	PC-3 cells	[150]
Induction of apoptosis via initiating the Fas death receptor/extrinsic pathway, which led to the dose-dependent upregulation of Bax, caspase-3, and caspase-9.	H522 lung cancer cells	[151]
Blocking transforming growth factor β and decreasing glucose uptake in a cancer cell by downregulating glucose transporter expression.	H441 lung cancer cells	[152]
Activation of the c-Jun-N-Terminal kinase (JNK) pathway, leading to reduction of cell viability and induction of apoptosis.	Human cancer cell line HCT-116	[157]
Elevation of Bax and caspase3, downregulation of the anti-apoptotic protein BCL-2, and induction of mitochondrial-mediated apoptosis.	Human cancer cell HT-29	[158]
Repression of the TGF-β1/Smad pathway and reduction of the levels of AST, ALT, hydroxyproline (Hyp), HA, LN, TNF-α, IL-6, extracellular matrix (ECM) production, and Smad2/3 phosphorylation.	HSC-T6 cells and male C57 mice	[160]
Reduction of the expression levels of ALP, ALT, AST, TGF-β1, HA, Hyp, F4/80þ macrophage infiltration, MCP-1, TNF-α, IL-1β, IL-6, TNF-α, and IL-1β, Gli-1, and Shh.	Littermate male C57BL/6J mice	[160]
Decreasing the expression of α-SMA, Col1α1, Col3α1, TIMP-1, PAI-1, and Gli-1 and increasing the levels of Bax and Caspase-3 that results in apoptosis.	LX-2 liver cells	[161]
Inhibition of cells migration, decrease cell viability, and induction of apoptosis via obstructing the intracellular signalling, including focal adhesion kinase (FAK), p38 phosphorylation, and caspase-3 activation.	Miapaca-2, Panc-1, and SNU-213 pancreatic cancer cell lines	[129]
Reduction of renal fibrosis, normalising renal function, and decreasing the expression of fibronectin (FN), Collagen I, α-SMA, EMT, Shh, Gli-1, and E-cadherin.	Renal cancer (NRK)-52E cell line and UUO-mouse model	[163]
Induction of apoptosis through a dose-dependent increase in the Bax/Bcl-2 ratio, cyt-c, caspase-3, caspase-9, AIF, and Apaf-1 via a mitochondrial-dependent mechanism.	Gastric cancer cells	[156]
Reduction of cell migration and invasion by inhibiting the expression of genes linked to the metastasis and lowering disruptor of telemetric silencing 1-like (DOT1L) and histone H3K79 methylation by controlling CBP activity.	Gastric cancer cells	[164]
Reduction of cell viability by decreasing Bcl-2 and raising Bax protein expression, thus inducing apoptosis, in addition to cell cycle arrest by decreasing cyclin B1 CDK1 and enhancing tumour suppressor gene p21 activation p38 MAPK, which arrests the G2/M phase.	U251 and U87 glioblastoma cells	[131]
Prevention of tumour growth via activating caspase-3 and -9, raising the Bax/Bcl-2 ratio, which caused apoptosis, and downregulation of the HIF-1α, VEGF, and VEGFR2 signalling pathway. In addition, decreasing the expression of cyclin B1 and D1 while increasing the expression of Claudin-1 and ZO-1 decreased the growth of cancer cells.	C6 glioma cells implanted in Wister rats	[169]
Induction of apoptosis via raising caspase-3 activity, MMP loss, and cell cycle arrest in the G2/M and G0/G1 phases.	HL60 leukaemia cell lines	[141]
Induction of apoptosis and cell cycle arrest at the G0/G1 phase, in addition to increasing the expression of the DUSP1 (dual specificity phosphatase 1), DUSP3, DUSP5, CDK1A, CDK1B GADD45B, SPRR2D, MT1F, MT1A, p27Kip1, CASP4, and NFKBIA genes. Moreover, elevation of the production of BAD, caspase-3, luciferase activity, PARP cleavage, and Notch 1 signalling.	K562 leukaemia cells	[140,170]
Increasing ROS generation, JNK1/2, p38, Bax, and p21 expression, while suppressing ERK1/2, cyclin B1, D1, D3, and E1 expression, which resulted in apoptosis and decreased cell viability.	A431 human cancer cells	[171]
Reduction of the expression of GSH, Bcl-2, and survivin while increasing the generation of ROS, cyt-c, caspase-9, -3, Apaf-1, Bax, and Sufu (suppressor of fused protein), in addition to decreasing PI3K/AKT signalling pathway, cyclin D1, MMP-2,9, and PI3K-p85 expression, as well as increased PTEN phosphorylation and p21 expression, which results in cell cycle arrest at the G0/G1 phase.	Oesophageal Eca-109 cancer cells	[173]

#### 5.1.6. Isoflavones

Isoflavones are among the most prevalent categories of phytoestrogens. It is mostly found in the Fabaceae family. These secondary plant metabolites are structurally identical to 17β-estradiol and are typically conjugated to it. They are hydrolysed into aglycones before being metabolised by the enzymes of the gastrointestinal tract or the microbiota found in the digestive tracts of humans and animals. Soy and its derived products are the primary sources of isoflavones [185]. Chickpeas and beans are additional dietary sources of isoflavones, and other plant products, such as fruits, vegetables, and nuts, also contain trace levels of isoflavones [186]. Genistein (Figure 2) is a naturally occurring phytoestrogen and isoflavone found in soybeans. Genistein has been shown to have numerous biological effects, including anti-oxidation, anti-proliferation, and tumoricidal properties [187] (Table 6). Isoflavones have significant antioxidant activity in addition to their oestrogenic activity. Two hydroxyl groups, such as those found in daidzein, must be present for antioxidant activity to occur (in the C-4 and C-7 positions). Compared to glycosides, aglycon molecules have higher activity [188]. Genistein exhibits strong antiproliferative properties against different cancer cells in vitro, inhibits the growth of tumours and shows an antimetastatic effect in vivo [189,190].

Compared to Western countries, where the average daily consumption of isoflavones is less than 2 mg, the incidence of breast cancer is lower in Asia, where the average daily intake of isoflavones approaches 25–50 mg [190]. Increased soy intake is associated with a lower risk of breast cancer [191].

Genistein inhibits breast cancer cell growth by promoting apoptosis [192,193]. Genistein is a prospective treatment for breast cancer since it acts as a weak oestrogen by binding to the oestrogen receptor. This could prevent the effects of natural oestrogens and slow the growth of breast cancer without causing any noticeable adverse effects [192,193,194]. α-ER activation promotes cell proliferation in breast tissue, whereas β-ER induces apoptosis and inhibits of cell proliferation. The precise ratio of α-ER to β-ER in cells determines how isoflavones affect the suppression or activation of cell growth [195].

In MDA-MB-231 cells, genistein has been shown to have antiproliferative properties, including the inhibition of NF-kB pathways and the subsequent inhibition of NF-κB [196]. Modification of the EGFR/Akt/NFκB pathway contributes to cell differentiation, ultimately resulting in the death of cancer cells. Genistein suppresses Akt activity, which encourages the deactivation of downstream signalling pathways such as NF-κB [197]. Moreover, it has been observed that genistein therapy reduces the expression of MMPs 2, 3, 3, and 15 in T47D cells, inhibiting angiogenesis and metastasis [198].

Genistein treatment of MCF-7-C3 and T47D breast cancer cells resulted in dysregulation of the human oncoprotein known as the carcinogenic inhibitor of protein phosphatase 2A (CIP2A), suggesting that CIP2A is a target of genistein responsible for inducing apoptosis and growth suppression [192].

In LNCaP and DU145 prostate cancer cell lines, both genistein and daidzein reduced cell growth and triggered apoptosis. The cuprous chelator neocuproine and other ROS scavengers, such as superoxide dismutase, catalase, and thiourea, dramatically reduced cell death induced by isoflavone. Copper chelation suppressed ROS production, supporting the idea that isoflavone-induced intracellular copper mobilisation leads to the production of ROS, which causes pro-oxidant cell death [199].

Phase I and II clinical trials on different cancers, including prostate cancer revealed that genistein inhibits metastasis of malignancies [200]. In addition, a significant decrease in serum PSA levels was observed in a prostate cancer trial [201].

**Table 6:** The anticancer effect of genistein in different tumour cells.

Anticancer Effect of Genistein	Cancer Type	Citation
Inhibition of NF-kB pathways and cell proliferation.	MDA-MB-231	[196]
Suppression of Akt activity, which encourages the deactivation of downstream signalling pathways, such as NF-κB.	MDA-MB-231	[197]
Reduction of the expression of MMPs 2, 3, 3, and 15, in addition to inhibition of angiogenesis and metastasis.	T47D cells	[198]
Dysregulation of the human oncoprotein, known as the carcinogenic inhibitor of protein phosphatase 2A (CIP2A), results in the induction of apoptosis and growth suppression.	MCF-7-C3 and T47D breast cancer cells	[192]
Induction of intracellular copper mobilisation leads to the production of ROS, which causes pro-oxidant cell death.	Prostate cancer cell lines LNCaP and DU145	[199]
Reduction of serum levels of prostate-specific antigen (PSA).	Prostate cancer cell lines	[201]

### 5.2. Phenolic Acids

**Figure 4:**
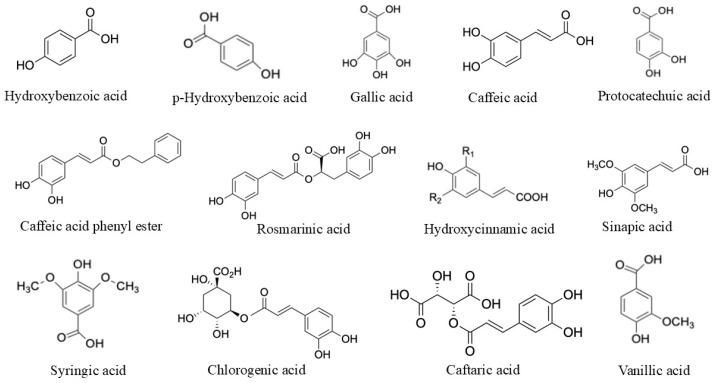
Structural identification of phenolic acids.

One of the most prevalent non-flavonoid plant phenolic components is phenolic acid (Figure 4), which can be found as glycosides or aglycones (free form) [202]. Phenolic acids are secondary metabolites that are widely distributed in plants [203] and are also present in oilseeds, grains, legumes, fruits, vegetables, herbs, and drinks [202]. Based on the C1–C6 and C3–C6 skeletons, phenolic acids are classified into two classes: hydroxybenzoic and hydroxycinnamic acids [204]. Gallic, Protocatechuic, *p*-Hydroxybenzoic, Syringic, and Vanillic acids are examples of hydroxybenzoic acids [202]. They mostly occur as conjugates. The Apiaceae family of spices and herbs has been found to have the highest concentration (fresh weight) of benzoic acids: anise (730–1080 mg kg^−1^), cumin (42 mg kg^−1^), fennel (106 mg kg^−1^), and parsley (30 mg kg^−1^) [203]. In contrast, P-coumaric, ferulic, caffeic, cinnamic, chlorogenic, and sinapic acids are examples of hydroxycinnamic acids. Plants contain large amounts of cinnamic acid in the form of amides or esters. Cereals, coffee, tea, wine, chocolate, fruits, and vegetables contain high levels of cinnamic acid. Wild blueberries (1470 mg kg^−1^), coffee (870 mg kg^−1^), carrots (260 mg kg^−1^), plums (234 mg kg^−1^), and eggplant (210 mg kg^−1^) are among the most significant sources of caffeic acid. Caftaric acid, a characteristic polyphenol found in wine, is one of the most significant derivatives of caffeic acid, and coffee contains significant amounts of chlorogenic acid [205,206]. By modulating several signalling pathways, hydroxycinnamic and hydroxybenzoic acids, as well as their derivatives, exhibit strong antioxidant and antiproliferative properties both in vitro and in vivo [207,208].

#### 5.2.1. Gallic Acid

Gallic acid (GA) (3,4,5-Trihydroxybenzoic acid) is a phenol obtained from plants that can inhibit the development and progression of different malignancies [209]. The strong anticancer effect of gallic acid may be due to its remarkable antioxidant activity. In addition, GA inhibits cancer cell invasion by inducing apoptosis in cancer cells [210]. Because of its antioxidant properties, GA exerts strong anticancer effects. It has been reported it improved the anticancer efficacy of docetaxel, cisplatin, doxorubicin, 5-FU, and paclitaxel in combination with gamma irradiation in vitro in a recent study using oral squamous cell carcinoma (FaDu and Cal33) cell lines. This was achieved through the superoxide-mediated apoptosis pathway, which is powered by lipophagy inhibition via the NRF2-dependent signalling pathway [211]. In a dose- and time-dependent manner, GA suppressed the growth and induction of non-small cell lung carcinoma (NSCLC) A549 cell line, which was linked to downregulated B-cell lymphoma 2 (Bcl-2) and increased (Bcl-2)-associated X protein (Bax) [212]. Upregulation of p53 (tumour suppressor protein) caused suppression of the PI3K/Akt pathway. This, in turn, regulated intrinsic apoptotic proteins like Bcl-2 and Bax and cleaved caspase-3 and cell cycle-related proteins like p27, p21, Cyclin E1, and Cyclin D1.

One of the main bioactive compounds in *Dovyalis caffra* (*D. caffra*) is GA. Specifically, it was discovered that at 1000 µg/mL, the plant’s methanol extract had 58.90% toxicity against HepG2 cells, suggesting potential anticancer properties [213]. Interestingly, GA played a crucial role in inducing ferroptosis in HepG2 cells. By preventing β-catenin transport from the nucleus to the cytoplasm, GA inhibits the production of ferroptosis-related proteins SLC7A11 and GPX4 in HepG2 cells. Thus, GA is a novel HC ferroptosis inducer, implying that GA may be a good candidate for the clinical treatment of hepatocellular carcinoma (HCC) [209]. By interacting with G-quadruplexes, GA has the potential to be a promising agent for cancer prevention, as demonstrated by Sanchez-Martin et al. [214]. Moreover, the findings demonstrated that nucleolar stress and the downregulation of G4-containing genes were caused by GA-induced cell cycle arrest in the S and G2/M phases.

However, GA has limited therapeutic utility because of its low oral permeability. 4-methoxybenzenesulfonamide (MBS), 3,4-dimethoxybenzenesulfonamide (DMBS), and 3,4,5-trimethoxybenzenesulfonamide (TMBS) were synthesised as GA analogues. To improve oral permeability and hydrophobicity, different quantities of methoxy groups, which are stronger electron-donating groups, were substituted for hydroxyl groups in these new compounds. Furthermore, a sulfonyl group, a more potent electron-withdrawing group, was substituted for the carboxylic group to boost the molecular polarity and antioxidative properties of the compounds. Compared to GA, TMBS was more successful in reducing DNA damage in lung cancer patients and PBMCs (peripheral blood mononuclear cells) healthy donors. Moreover, TMBS was more cytotoxic to A549 cells, while it did not cause cytotoxicity in healthy PBMCs, indicating that TMBS may have therapeutic value in cancer treatment [210].

#### 5.2.2. Caffeic Acid

It is commonly known that natural caffeic acid (E)-3-(3,4-Dihydroxyphenyl) prop-2-enoic acid has several biological characteristics, including anticancer effects. Min et al. found that caffeic acid (CA) causes apoptosis, which dramatically reduces the proliferation of H1299 NSCLC cells [215]. When CA and paclitaxel (PTX) were combined, they exhibited a synergistic anticancer effect on H1299 cells. This combination inhibited the proliferation of H1299 NSCLC cells. CA administration enhanced H1299 cell apoptosis, caspase-3, and caspase-9 activity, and sub-G1 phase arrest. It also enhanced PTX-induced activation of Bid, Bax, and downstream of PARP cleavage. Moreover, it elevated the phosphorylation of kinase1/2 and c-Jun NH2-terminal protein kinase1/2. Lipid hydroperoxides, reactive thiobarbituric acid substances, and connective dienes are indicators of lipid peroxidation that are elevated by CA. Additionally, it enhanced morphological alterations, changed the potential of the mitochondrial membrane, and increased ROS levels in cells treated with CA, indicating that CA has anticancer activity because of its pro-oxidant function [216]. A study conducted by Rosendahl et al. revealed that CA mimics anti-oestrogen action and modifies important growth regulatory signals, including ER/cyclin D1 and IGF-IR/p-Akt, resulting in cell cycle damage and reduced cell proliferation [217].

To assess the effect of CA on the toll-like receptor 4 (TLR4) signalling pathway, Chen et al. conducted research that showed that CA lowered the production of IL-12 and NF-κB activation. However, by changing the TLR4/MD2 complex, the TLR4 pathway was hindered. These findings demonstrated that apoptosis in breast tumours is caused by the downregulation of TLR4, TRIF, and IRAK4 expression [218].

Furthermore, by inhibiting ERK phosphorylation, CA dramatically reduced lung metastasis caused by CT-26 colon cancer cells. Additionally, CA is directly bound to MEK1 or TOPK and significantly suppresses mitogen-activated MEK1 and TOPK activities. CA inhibited the neoplastic transformation of JB6 P+ cells, AP-1 and NF-κB transactivation, and ERK phosphorylation induced by EGF and H-Ras [219]. Moreover, Yang et al. reported that both in vitro and in vivo models showed that CA efficiently decreases tumour incidence and volume as well as colony formation. The CA-treated mouse skin cancer xenograft model showed a reduction in MAPK phosphorylation. Furthermore, CA directly interfered with ERK1/2 and inhibited ERK1/2 activity in vitro. It also interacts with ERK2 at the Q105, D106, and M108 amino acid residues [220]. High chemopreventive effects against A549 lung adenocarcinoma cells were shown for CA phenethyl ester, a CA derivative that represents a naturally occurring phenolic chemical that is found abundantly in plants and propolis extract, in the context of lung cancer. It was extremely important in reducing TGF-β-promoted cell motility and changing the growth factor-β (TGF-β)-induced activation of Akt (protein kinase β) and blocking the phosphatidyl inositol 3-kinase (PI3K)/Akt pathway.

Research on prostate cancer has revealed that CA phenethyl ester can prevent NF-κB activation in prostate cancer-3 (PC-3) cells by preventing TNF-α and Paclitaxel from activating NF-κB. Nevertheless, this action is also associated with decreased levels of apoptosis-inhibiting proteins (cIAP1, cIAP-2, and XIAP) in cells, as well as the downregulation of elevated levels of spontaneous apoptosis and cIAP-1 expression [221]. Moreover, by controlling Skp2, p53, p21Cip1, and p27Kip1, CA phenethyl ester has been reported to cause cell cycle arrest and growth suppression in castration-resistant prostate cancer cells [222].

Amorim et al. showed that while ROS elimination was unsuccessful, AntiOxCIN6 (a mitochondria-targeted antioxidant) enhanced the antioxidant defence system in HepG2 cells. AntiOxCIN6 markedly affected mitochondrial structure and function, which led to a reduced ability to produce complex I-driven ATP without affecting cell viability. Glycolytic flux increases in tandem with these changes [223]. They also mentioned that AntiOxCIN6 appears to produce metabolic alterations or redox pre-conditioning in lung MRC-5 fibroblasts, protecting cisplatin, while it sensitises A549 adenocarcinoma cells for CIS-induced apoptotic cell death. They suggested that the length and hydrophobicity of the C10-TPP+ alkyl linker are important factors in causing cellular and mitochondrial toxicity, whereas the antioxidant caffeic acid seems to oversee triggering cytoprotective mechanisms.

When paired with anticancer medications, caffeic acid increased apoptosis and suppressed the growth and clonogenicity of acid-adapted cancer cells by inhibiting the hyperactivation of the PI3K/Akt and ERK1/2 signalling pathways linked to drug resistance. Thus, its potential for overcoming drug resistance in cancer therapy is highlighted by its capacity to suppress proliferation, sensitise cells to apoptosis, and alter the signalling pathways [224].

In contrast, a combination of gamma-cyclodextrin (γCD) with CA phenethyl ester was also found to exert cytotoxic effects on several cancer cells [225]. The strong anticancer and antimetastatic effects of this complex were suggested to occur via the disruption of mortalin-p53 complexes, resulting in p53 nuclear translocation and activation, leading to the growth arrest of cancer cells [226].

Further research was performed on pancreatic ductal adenocarcinoma (PDAC) cell lines to investigate the pre-sensitising effects of CA in combination with several medications. In Panc-1 and Mia-PaCa-2 PDAC cell lines, CA pre-sensitisation decreased the doxorubicin IC50 concentration, which also caused ROS production. Following CA treatment, differential gene expression analysis revealed that distinct genes were affected in both cell lines, including p53 and Pi3K/Akt/mTOR in Mia-PaCa-2 cells and N-Cad and Caspase-9 in Panc-1 cells [227].

Comparing the effect of CA phenethyl ester (IC25 = 1.3 μM/IC50 = 2.7 μM) to CA alone (IC25 = 91.0 µM/IC50 = 120.0 µM), it was found that CA phenethyl ester reduced mitochondrial ROS generation, cell migration, and cell survival in murine osteosarcoma UMR-106 [228].

All proposed derivatives of caffeic acid were subjected to molecular docking investigation, which focused on the three-dimensional coordinates of human DHFR (PDB ID 1U72) co-crystallised with methotrexate (MTX). In addition to their anticancer and antibacterial properties, a new series of 1,2,4-triazole analogues of caffeic acid were developed, synthesised, characterised, and evaluated for their ability to inhibit DHFR [229].

#### 5.2.3. Rosmarinic Acid

Rosmarinic acid (RA) is present in 39 plant families [230]. It is particularly present in many species of the Nepetoideae subfamily of the Lamiaceae family and the Boraginaceae family [231,232]. Despite being widely found in the plant kingdom, rosmarinic acid is the only significant chemotaxonomic marker of the Lamiaceae family [233]. Plants in the Lamiaceae family, including rosemary, produce rosmarinic acid (RA) as a secondary metabolite. Rosmarinic Acid (2R)-3-(3,4-Dihydroxyphenyl)-2-{[(E)-3-(3,4-dihydroxyphenyl) prop-2- enoyl]oxy} propanoic acid is one of the esters of caffeic acid [234].

Numerous pharmacological properties of RA have been identified, including antibacterial, antiviral, antimutagenic, antioxidant, and anti-inflammatory activities [232,235]. Furthermore, RA functions as a neuroprotective and immunomodulatory factor [236]. Additionally, its ability to inhibit tumour growth has been observed in a variety of cancer types, including colon, breast, liver, stomach, lung, melanoma, and leukaemia [233,237].

In human oral cancer cells, reduction of cancer cell migratory capacity, activated of apoptosis, induced cell cycle arrest at the G2/M phase, and inhibiting of cell proliferation in a dose-dependent manner [238]. In gastric adenocarcinoma cells, RA reduced the activity of MMP-9, which is crucial for cancer spread because it breaks down collagen and other extracellular matrix proteins [239]. In the WiDr colon cancer cell line, apoptosis was activated, and RA showed antiproliferative effects. Caspase 1 and Caspase 7, which are essential for apoptotic pathways, were upregulated, while BCL2 was downregulated [240]. Additionally, through the regulation of the Nrf2/Keap1 pathway and modulation of miR-1225-5p, RA was able to prevent the migration and invasion of HT-29 colorectal cancer cells. Cellular defence against oxidative stress (OS) is significantly influenced by this pathway, and RA’s capacity of RA to inhibit p38/AP-1 signalling through IL-17RA targeting offers more proof of its anticancer potential [241]. Through its suppression of TLR4-mediated NF-κB-STAT3 signalling, which is essential for colon carcinogenesis and inflammation, RA decreased tumour incidence and inflammation in a mouse model [242]. In addition, by suppressing miR-155 and inhibiting hypoxia-inducible factor-1 alpha (HIF-1α), RA influences the IL-6/STAT3 pathway, reducing inflammation and encouraging cancer cell death [243]. In OC3 and DU145 prostate cancer cell lines, RA suppressed colony and spheroid formation, as well as cell proliferation. Moreover, RA therapy was able to successfully inhibit a histone deacetylase enzyme that controls the expression of mitochondrial intrinsic apoptotic pathway genes, such as Bcl-2, Bax, caspase-3, and poly (ADP-ribose) polymerase-1 (PARP-1). When RA downregulates HDAC2, a tumour suppressor protein called p53 is activated, which causes prostate cancer cells to undergo apoptosis. In addition, RA upregulates p21 expression and downregulates proliferating cell nuclear antigen, cyclin D1, and cyclin E1, resulting in apoptosis [244]. In addition to inducing apoptosis, RA dramatically reduced cell invasion, migration, and proliferation in DU-145 prostate cancer cell lines. RA shows promise in preventing the spread of cancer to other organs and improving patient outcomes in advanced prostate cancer by preventing the migration and invasion of cancer cells [245].

In contrast, RA is considered one of the important polyphenolic elements of *Glechoma hederacea* L.’s ethyl acetate fractionated extract (EAFE), which has been shown to preventing HepG2 cell proliferation, leading to apoptosis and cell arrest in the S phase. This EAFE’s apoptogenic activity involves Ca^2+^ buildup, ROS generation, MMP disruption, caspase 3, 9 activation, elevated Bax/Bcl-2 ratio, and glutathione depletion [246]. Under the same conditions, RA significantly inhibited the proliferation of SMMC-7721 cells and increased G1 arrest and apoptosis in a concentration-dependent manner. Furthermore, RA was able to inhibit cell invasion by controlling epithelial-mesenchymal transition and suppressing the PI3K/AKT/mTOR signalling cascade [247].

In U2OS and MG63 osteosarcoma cells, RA was able to inhibit DJ-1 expression by regulating the PTEN/PI3K/Akt signalling pathway. Moreover, DJ-1 has been suggested to be a biological target of RA in osteosarcoma cells. RA induced apoptosis by upregulating the cleavage rates of caspase-8, caspase-9, and caspase-3, thus enhancing the Bax/Bcl-2 ratio, which resulted in ROS generation and decreased matrix metalloproteinase (MMP) [248]. Due to its ability to alter many signalling pathways that result in the growth of tumour tissue, there is compelling evidence that RA may be a potential therapy for several BC types [230,249]. Potent antiproliferative effects and cytotoxicity of RA have been reported in a dose- and time-dependent manner in breast cancer cell lines. MDA-MB-231 cell underwent apoptosis and cell cycle arrest in the G0/G1 phase after RA treatment. RA significantly upregulated the expression of tumour necrosis factor receptor superfamily 25 (TNFRSF25), harakiri (HRK), and BCL-2 interacting protein 3 (BNIP3) while inhibiting the expression of TNF superfamily 11B receptor (TNFRSF11B). Moreover, RA was able to markedly activate TNF transcription and cause growth inhibition and DNA damage-inducible 45 alpha (GADD45A) and BNIP3 [236]. Furthermore, in MDA-MB-231 breast cancer cells, RA suppressed MARK4 (microtubule affinity-regulating kinase 4) activity, which led to dose-dependent apoptosis. RA successfully targeted MARK4, a kinase implicated in the progression of cancer, indicating that it may be a suitable therapeutic target for breast cancer. The MARK4 protein, which is closely linked to breast cancer, has a high affinity for RA. Their 500 ns all-atom simulations and molecular docking showed that RA forms stable non-covalent interactions with important residues in the MARK4 active site, indicating that RA may prevent MARK4 from playing a role in the development of cancer [235]. Additionally, RA has been reported to induce both apoptosis and autophagy and to show dose-dependent suppression of breast cancer cell proliferation, especially in oestrogen-dependent MCF7 cells [250]. Moreover, RA promotes apoptosis by upregulating Bax and downregulating Bcl-2 expression. This lends more credence to the theory that important apoptotic proteins, like Bcl-2 and Bax, are regulated by RA to cause apoptotic effects in breast cancer cells [251]. In addition, by decreasing matrix metalloproteinase-9 (MMP-9) activity, RA successfully prevented the invasion and migration of cancer cells. MMP-9 is a proteolytic enzyme essential for the disintegration of the extracellular matrix, which promotes the spread of cancer [252].

In contrast, the RA-loaded microemulsions exhibited superior antioxidant activity compared to free RA in breast cancer cells (T47D and MDA-MB-231). Furthermore, they induced cell cycle arrest apoptosis, and inhibited cell growth. The greater bioavailability and stability of RA when administered via microemulsions have been credited with this increased therapeutic efficacy [253].

Regarding in vivo research on breast cancer models in mice has shown that RA exhibits antitumor activity alone or in combination with paclitaxel. VEGF, TNF-α, and NF-kB were repressed after RA treatment, while Bcl-2, p53, Bax, and caspase-3 were restored, leading to apoptosis. In addition, inhibition of tumour growth with elevated p53 and caspase-3 and repressed Bcl2/Bax ratio was observed in Ehrlich tumours in mice after RA administration, either alone or in combination with paclitaxel [254].

By blocking the ADAM17/EGFR/AKT/GSK3β pathway, RA prevents invasive proliferation and migration of human melanoma A375 cells, induces apoptosis, and increases the susceptibility of melanoma cells to cisplatin [255]. In pancreatic cancer cell lines (Panc-1 and SW1990), RA promoted apoptosis and inhibited cell viability, motility, and invasion. The study found that RA suppresses epithelial-mesenchymal transition (EMT), a crucial step in cancer metastasis, by upregulating miR-506 and suppressing MMP2 and MMP16 [256]. According to Zhou et al., RA was able to inhibit Gli1 nuclear translocation and induce Gli1 degradation by proteasomes. By blocking MMP-9 and E-cadherin, RA prevents cell invasion and migration [257]. Through the regulation of matrix metalloproteinases (MMP-2 and MMP-9) and the upregulation of E-cadherin expression, while downregulating N-cadherin and vimentin, RA also prevented the growth, invasion, and metastasis of hepatocellular carcinoma (HCC) cells in male BALB/c nude mice. By blocking the PI3K/AKT/mTOR signalling pathway, which is necessary for cancer cell survival and proliferation, RA decreases tumour volume and increases apoptosis rates [247].

In liver tumours, RA inhibits NF-κB signalling, thereby decreasing inflammation-related cytokines and angiogenesis. This suggests that RA acts similarly to how it inhibits HSP90AA1 in liver cancer [258]. 

A crucial chaperone protein, heat shock protein 90 (HSP90) interacts with oncogenic client proteins and co-chaperones to regulate signaling cascades and fix misfolded proteins in cancer cells [259]. The relationship between RA and HSP90AA1, a protein essential for the survival and growth of cancer cells in liver cancer, was examined using molecular docking and dynamics simulations. According to this study, RA generates strong hydrogen bonds at the active site of HSP90AA1 and binds to it with high affinity. This implies that RA may reduce cancer cell survival by blocking HSP90AA1’s carcinogenic activity [15]. Furthermore, RA therapy targeted genes implicated in tumour progression and aberrant cell proliferation and downregulated the oncogenic transcription factor forkhead box M1 (FOXM1). FOXM1 was also inhibited in ovarian cancer cells after treatment with a combination of cisplatin and RA methyl ester, which reversed cisplatin resistance [260]. In A549 lung adenocarcinoma cells, RA decreased OS, inflammation, and metastasis, involving pathways such as Akt, P-65-NF-κB, and c-Jun [261]. By inhibiting NF-κB activation and ROS production, RA made human leukaemia U937 cells more sensitive to TNF-α-induced apoptosis. In addition to reducing ROS levels, RA’s suppression of NF-κB increases caspase-dependent apoptosis activation, which lowers cancer cell survival [262]. By activating MAPK and inhibiting the expression of P-gp and MDR1, RA can inhibit the growth and cell colony formation, induce apoptosis and cell cycle arrest of non-small cell lung cancer (NSCLC) in a dose-dependent manner, in addition to elevating the sensitivity of cisplatin-resistant cells [263]. In non-small cell lung cancer (NSCLC), RA targets focal adhesion kinase (FAK). RA can bind to FAK to form stable complexes that block the signalling pathways linked to metastasis [264].

Bone metastases can be inhibited by RA. Therefore, RA could be a promising option for a new therapeutic approach for breast cancer bone metastases [265]. Additionally, ST-2 murine bone marrow stromal cells cultured with RA showed a significant and dose-dependent increase in alkaline phosphatase activity, in addition to an increase in the quantity and size of mineralised nodules. RA may prevent bone metastasis from breast cancer by simultaneously reducing the synthesis of interleukin-8 and the receptor activator of NF kappaB ligand (RANKL/RANK/osteoprotegerin) pathway [266]. As osteoprotegerin is a pro-angiogenic factor, its inhibition may help stop the spread of cancer cells. Moreover, increased IL-8 expression by breast cancer cells has been associated with osteolysis in metastatic breast cancer [254]. Furthermore, the decrease in human umbilical vein endothelial cell proliferation, adhesion, migration, and tube formation has demonstrated that RA has antiangiogenic properties, which aid in preventing cancer development and metastasis [267]. In human HaCaT keratinocytes, a model for UV-induced skin cancer, RA in conjunction with fucoxanthin showed that by upregulating Nrf2 and HO-1 and downregulating inflammasome components such as NLRP3 and Caspase-1, RA and fucoxanthin decreased UVB-induced apoptosis and inflammation. These results suggest that RA may have a preventive function against skin cancer, especially by reducing the negative effects of UV exposure [268]. RA reduces oxidative imbalance and mitochondrial fragmentation caused by UVB rays, which are two major factors in the development of skin cancer. By modifying mitochondrial dynamics and ROS levels, RA appears to shield skin cells from the harmful effects of UV radiation, suggesting a possible protective role for RA against sun-induced skin cancer [269]. Additionally, by activating the Nrf2 pathway, a crucial regulator of the cellular antioxidant response, RA increases the activity of antioxidant enzymes like SOD, CAT, and heme oxygenase-1 (HO-1) [270].

#### 5.2.4. Sinapic Acid

Sinapic acid (5-dimethoxy-4-hydroxycinnamic acid) can be extracted from different vegetables, fruits, cereals, spices, and oilseed crops [271]. Sinapic acid (SA) has anti-inflammatory and anti-apoptotic effects, neuroprotective, anti-inflammatory, anti-nociceptive, anti-allergic, and antioxidant properties [272], and the ability to scavenge free radicals [273]. Additionally, SA reduced intestinal inflammation in a mouse model of colitis, attenuated chemical reagent-induced clinical symptoms such as 2,4,6-trinitrobenzenesulfonic acid (TNBS) and DSS [274,275], and exhibited potent efficacy against bleomycin-induced pulmonary fibrosis in rats [276]. Furthermore, SA preserves epithelial homeostasis in lipopolysaccharide-induced Caco-2 cells and prevents inflammation-induced intercellular hyperpermeability [272].

SA was reported to inhibit histone deacetylase (HDAC), which resulted in ROS release, oxidative stress, apoptosis, cell cycle arrest, and autophagy [277]. Moreover, by downregulating the AKT/Gsk-3β pathway, SA was able to inhibit the proliferation, migration, and invasion of pancreatic malignant cells [278]. Furthermore, in vitro studies on lung cancer cells revealed elevated ROS and caspase-3 levels by SA, resulting in cytotoxicity and apoptosis, while lung cancer in vivo studies exhibited a reduction in IgG and IgM, leucocytic count, and tumour markers, with improved phagocytic activity and enzymes involved in antioxidant defence [279]. In addition, elevated ROS production, apoptosis, and cell cycle arrest at the G0/G1 phase were observed after exposure of the HEp-2 human laryngeal carcinoma cell line to SA [280].

Exposure of prostate cancer cell lines to SA revealed its antiproliferative and cytotoxic effects. LNCap cells showed significantly elevated levels of caspase-3, caspase-9, CYCS, and Bax, with a marked reduction in CDH2, MMP-2, and MMP-9. PC-3 cells exhibited caspase-3, caspase-8, Bax, CYCS, TMP-1, FAS, and CDH1 expression after SA treatment, with a significantly lower level of MMP-9 [281]. In vivo, research on DMH-induced colon carcinogenesis showed an increase in antioxidant defence through elevated superoxide dismutase (SOD), catalase (CAT), and glutathione peroxidase (GPX) [282].

In contrast, the combination of SA with cisplatin strongly inhibited the migration and proliferation of hepatocellular carcinoma cells by inducing apoptosis and autophagy [283]. The complex of SA with CYP3A4, CYP1A1, and SIRT1 proteins was studied using molecular dynamic simulations and MMPBSA, which showed a stable complex over the course of the simulations. According to these predictions, the mechanism of SA in breast cancer may involve the regulation of several proteins, including cytochrome enzymes (CYP1A1 and CYP3A4), PRKCA, CASP8, SIRT1, and CTNNB1. Interestingly, MDA levels were significantly elevated, SOD activities were significantly reduced, and reduced glutathione (GSH) and catalase levels were elevated in MCF-7 cells treated with sinapic acid-loaded poly (lactic-co-glycolic acid) (PLGA) nanoparticles (SaNPs) at concentrations of 150 and 200 μg/mL for 24 h compared to the control groups [284].

### 5.3. Curcuminoids: Curcumin

**Figure 5:**
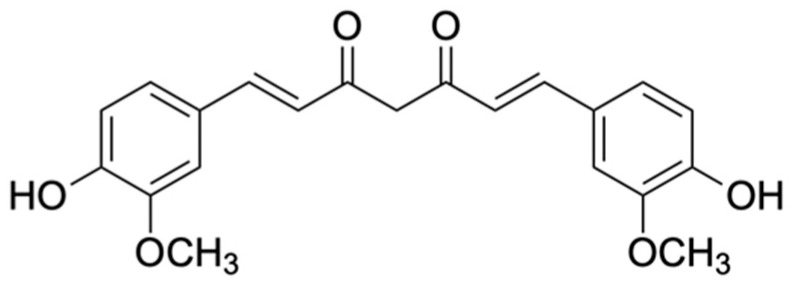
Chemical structure of curcumin.

Curcumin (Figure 5) is the active ingredient in turmeric. Demethoxycurcumin and Bis-demethoxycurcumin, two curcuminoids found in the yellow-pigmented fraction of Curcuma longa, are chemically related to curcumin, the plant’s main ingredient. In aqueous solutions, curcumin (CUR) dissolves very little or not at all; nevertheless, it dissolves in organic solvents such as acetone, ethanol, methanol, and dimethyl sulfoxide (DMSO) [285]. Curcumin has a symmetrical structure with four chemical entities: aryl side chains joined by a linker, two double bonds, an active methylene moiety, and a diketo functional group. Each of these sites has been studied to identify possible locations for appropriate changes to enhance the solubility, bioavailability, and effectiveness of curcumin [286].

Curcumin exhibits potent antiproliferative and antimetastatic properties, in addition to its antioxidant and pro-apoptotic activities, in various cancer cell lines [287]. Several in vitro studies have repeatedly demonstrated curcumin’s strong antioxidant properties [288,289,290]. By scavenging free radicals and boosting endogenous antioxidant defences, it can reduce oxidative stress [290]. Additionally, curcumin’s anti-inflammatory properties have been clarified by in vitro research, which has shown that it inhibits the generation of proinflammatory mediators and modifies important inflammatory pathways. Curcumin and its analogues have demonstrated therapeutic potential in numerous preclinical models of inflammation-associated diseases, such as arthritis, colitis, and neuroinflammatory disorders [285]. Furthermore, curcumin’s anticancer properties have been supported by both in vitro and in vivo research. These effects include inhibition of angiogenesis, induction of apoptosis, suppression of tumour growth, and modification of several signalling pathways implicated in carcinogenesis [291,292]. Furthermore, curcumin exhibits synergistic effects with some anticancer medications, providing a promising approach to treating cancer [293].

It has been reported that curcumin exerts an anticancer effect in human and mouse MM cells in a dose- and time-dependent manner by increasing ROS production, inducing DNA damage and apoptosis, and inhibiting cell survival and growth. PARP-1 cleavage, p53, Caspase-9, and Bax/Bcl-2 ratios were also elevated. Moreover, it stimulated ERK1/2 and P38 MAPK phosphorylation and increased c-Jun expression and phosphorylation while inhibiting P54JNK and AKT phosphorylation and the nuclear translocation of NF-kB [294]. In addition, curcumin can reduce the activity of IkB kinase, thus retarding the degradation of IkBα, which leads to the inhibition of the nuclear translocation of NF-kB [295].

In contrast, curcumin was able to suppress different inflammatory cytokines, including TNF-α, IL-6, IL-8, and IKKβ kinase in head and neck squamous cell carcinoma, in addition to its ability to inhibit protein kinases such as PKA, mTOR, PhK, and MAPK that are essential for regulating cell survival, proliferation, and growth [296]. Moreover, curcumin was able to induce oxidative stress, apoptosis, autophagy, and cell cycle arrest in human glioblastoma by modulating different pathways [297]. In a dose-dependent manner, curcumin inhibited the transcription factor NF-kB in GBM8401 cells via the induction of caspase and mitochondrial-dependent apoptosis [298].

Gefitinib’s antitumor activity in the xenografted NSCLC cell lines and mouse model was enhanced by curcumin, which suppressed NSCLC proliferation, EGFR phosphorylation, EGFR ubiquitination, and induced apoptosis [299]. Furthermore, curcumin inhibited JAK2 activity in A549 human lung cancer cell lines, downregulating NF-κB activity and acting on the JAK2/STAT3 signalling pathway. Curcumin is effective in treating lung cancer [300].

Curcumin was able to suppress clonogenicity cell proliferation and induce cell cycle arrest at the G2/M phase in leukaemic cell lines via dose-dependent inhibition of Wilms tumour protein 1 (WT1). Furthermore, by inhibiting EZH2 expression in RPMI8226 and U266 cell lines, curcumin potently suppressed MM cell growth by inducing apoptosis in a time- and dose-dependent manner [301].

Many signalling pathways linked to breast cancer, such as JAK-STA, Hedgehog, Notch, PI3K/mTOR, and Wnt/β-Catenin, were successfully targeted by curcumin [302]. Moreover, it can inhibit the growth of breast cancer cells via DNA methylation and epigenetic changes [303]. Curcumin inhibited the proliferation of MCF-7 breast cancer cells by arresting them in the G1 phase of the cell cycle. This cell cycle arrest was due to the overexpression of CDK inhibitors p21, p53, and p27, as well as increased cyclin E proteasomal degradation [304]. Moreover, by suppressing EZH2 and re-establishing DLC1 expression, CUR suppressed the growth of TNBC and enhanced apoptosis in MDA-MB-231 cells during the G2 phase [305].

Curcumin enhanced the cytoprotective effect and stability against HepG2 cell death induced by tert-butyl hydroperoxide (t-BHP) and facilitated the nuclear translocation of transcription factor Nrf-2, which regulates the antioxidant signalling pathway [306]. Curcumin also targeted and disrupted the intracellular Notch domain of the Notch-1 signalling pathway in HEP3B, SK-Hep-1, and SNU449 cell lines. Additionally, curcumin prevents diethylnitrosamine (DENA)-induced hyperplasia and HCC in animals by lowering the expression of p53, NF-κB, and p21-Ras [307]. A combination of arabinogalactan and curcumin significantly inhibited the proliferation of breast cancer cells without affecting normal cells. This combination induces cell apoptosis by altering membrane potential, increasing ROS levels, and lowering glutathione levels. Additionally, by overexpressing p53 in mice, the combination of curcumin and arabinogalactan prevented the growth of breast tumours [308]. Interestingly, several studies have suggested that the co-treatment of CUR with conventional chemotherapy drugs produces varying levels of efficacy in BC cells compared to normal epithelial cells [309]. In MDA-MB-231, MCF-7, and MCF10A cells, CUR and doxorubicin (DOX) treatments caused G2/M arrest; however, in MCF10A cells, CUR caused S phase arrest [310].

Luo et al. investigated the antiproliferative effects of four curcumin analogues on human gliomas. Curcumin (IC50 = 4.19 µM), bisdemethoxycurcumin (IC50 = 29.15 µM), demethoxycurcumin (IC50 = 30.03 µM), and dimethoxycurcumin (IC50 = 29.55 µM) were the four analogues that were most promising in promoting sub-G1 phase, G2/M arrest, apoptosis, and ROS production in human glioma cells. Dimethoxycurcumin inhibited migration, colony formation, and cell viability; it enhanced LC3B-II expression to trigger autophagy, a natural process that preserves cellular health by dissolving and recycling damaged or unnecessary components; and it caused sub-G1, G2/M arrest, apoptosis, and ROS production. They also examined several curcumin analogues for antitumor properties against the human breast cancer cell (Bcap-37), prostate cancer cells (PC3), and gastric cancer cell line (MGC-803). One of the chemicals was less harmful to NIH3T3 normal cells, while dramatically reducing the development of cancer cells and inducing apoptosis in MGC-803 cells [311].

Due to their high methylation, unsaturated diketone moiety, and low hydrogenation, several curcumin derivatives have demonstrated improved anticancer and anti-inflammatory properties compared to curcumin. Furthermore, numerous analogues of hydrogenated curcumin have also demonstrated strong antioxidant activity [312]. Moreover, novel drug delivery systems have been investigated to improve the stability of curcumin by increasing its absorption and bioavailability. These systems include nanoparticles, metal complexes, liposomes, solid dispersion, microemulsion, micelles, nanogels, and dendrimers [313]. Curcumin-containing catanionic lipid nanosystems (CLNs) have been observed to exhibit improved cytotoxicity against Lewis lung cancer cells [314].

It has been proposed that curcumin and its derivatives enhance BC cells’ defence mechanisms by stopping the cell cycle. Every phase contributes to the development of cancer; however, because of its beginning position and function—cell duplication—the G1 phase is frequently seen as being especially important in fostering the development of cancer. Solid lipid nanoparticles (SLNs) loaded with CUR stop the cell cycle at G1/S and reduce the production of CDK4 and cyclin D1 (CCND1), which potently triggers ROS responses and apoptosis [315]. Moreover, the CUR analogue B14 modifies the expression of cyclin D1 (CCD1), cyclin E1, and cyclin-dependent kinase 2 (CDK2), causing G1 phase cell cycle arrest and initiating the mitochondrial apoptotic pathway [316]. Nonetheless, the G2/M phase is where most CUR combinations stop the cell cycle. When CUR was combined with a layered polyelectrolyte capsule, the number of cells in G2 increased significantly. Consequently, the proportion of apoptotic cells increased noticeably [317]. Additionally, MCF7 cells were more significantly affected by CUR, berberine, and a combination of 5-FU loaded into nano micellar particles at lower doses [318]. Moreover, gemcitabine and CUR can be combined as a nanosuspension to increase their anticancer potentiality in a synergistic manner [319].

Disruption of microtubule assembly by mesoporous silica nanoparticles can affect the cell cycle. CUR-MSN-polyethyleneimine (PEI)-FA was more successful in causing the G2/M phase cell cycle arrest by comparing the efficiency of CUR-MSN-HA and CUR-MSN-PEI-FA in MDA-MB-231 and MCF-7 cell lines [320]. In MCF-7 cells, it was demonstrated that the CUR analogue (2E,6E)-2,6-bis-(4-hydroxy-3 methoxybenzylidene)-cyclohexanone (BHMC) stimulates apoptosis and G2/M cell cycle arrest [321].

Co-encapsulation of doxorubicin and curcumin in chitosanpoly (butyl cyanoacrylate) nanoparticles has been shown to reverse multidrug resistance (MDR) [322]. Solid lipid nanoparticles (SLNs) loaded with CUR avoided P-pg MDR in TNBC cells [323]. To reverse multidrug resistance in breast cancer, doxorubicin, and curcumin were delivered using amphiphilic copolymeric micelles [324]. Curcumin reduces doxorubicin resistance in doxorubicin-resistant breast cancer cells by blocking ABCB4’s efflux activity [325]. Moreover, the combination of CUR with DOX markedly enhanced apoptosis in proliferative MCF7 cells compared to DOX treatment alone [326].

According to Liu et al., curcumin has been shown to have beneficial effects in clinical trials; nevertheless, it may also have adverse effects on the heart, liver, kidneys, blood, reproductive system, and immune system [327]. Curcumin’s efficacy in treating a variety of malignancies, including colorectal cancer, myeloma, oral submucosal fibrosis, and skin lesions, has been investigated in clinical trials [285]. Salehi et al. reported histological improvements and clinical alleviation in individuals with skin lesions and oral submucosal fibrosis after curcumin treatment. Additionally, in individuals with various malignancies, curcumin showed tumour growth reduction and downregulation of inflammatory markers; nevertheless, its effectiveness in treating advanced pancreatic cancer remains restricted [328].

Preclinical research on curcumin’s antioxidant, anti-inflammatory, and anticancer properties, as well as those of its analogues, has yielded intriguing therapeutic prospects for a range of diseases. However, further clinical research is required to validate curcumin’s toxicity, bioavailability, and effectiveness [285].

### 5.4. Stilbenes

**Figure 6:**
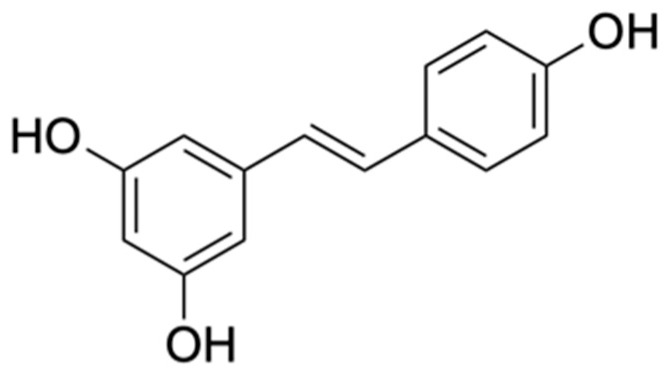
Chemical structure of resveratrol.

Resveratrol (RSV) (Figure 6) is a naturally occurring phytoalexin produced by plants to defend against pathogenic invasion and environmental stress, and it may help treat cancer and signal advances in cancer treatment. Although it can be found in over 70 plant species, the most significant sources are grapes and wines [329].

There are two isomers of resveratrol. Due to its instability, the cis-isomer is not marketed. Although its trans-isomer is more stable, it degrades more quickly when exposed to high pH or UV light, changing into the cis-isomer. The trans-isomer is thought to be the cause of resveratrol’s anticancer and health effects [330]. Resveratrol is crucial for increasing immunity, delaying the ageing process, and imitating the effect that prevents or lessens diseases such as diabetes, in addition to cardiovascular and neurodegenerative diseases [331]. Resveratrol’s direct antitumor, anti-inflammatory, and antioxidant properties make it a promising agent for conventional chemotherapy [332].

It has demonstrated effectiveness against lung, skin, and haematological cancers, as well as obesity-related cancers like hepatic, pancreatic, postmenopausal breast, prostate, and colorectal cancers [333].

It has been demonstrated that resveratrol inhibits the plasma levels of insulin-like growth factor-1 (IGF-1) and insulin-like growth factor-binding protein-3 (IGFBP-3), two proteins involved in the insulin signalling pathway that cause carcinogenesis [334]. Additionally, its therapy decreased prostaglandin-E2 (PGE2) production and Ras-association domain family-1a (RASSF-1a) methylation, both of which are associated with antiproliferative and anti-inflammatory effects [335]. Moreover, when resveratrol was administered, immunomodulatory T cell levels were significantly upregulated, proinflammatory cytokines like monocyte chemoattractant protein-1 (MCP1) and tumour necrosis factor-alpha (TNF-α) were downregulated, and plasma antioxidant activity was higher than the baseline [336]. Furthermore, resveratrol demonstrated an antiproliferative effect by blocking wingless-related integration site (Wnt)signalling [337]. Overall, low dosages of resveratrolseem to have chemopreventive ability based on its effects on specific tumour markers [330].

In contrast, pterostilbene (PTE), a naturally occurring resveratrol analogue [338], is abundant in blueberries [339]. Pterostilbene has attracted considerable interest because of its potential medical applications in the treatment of cancer and inflammatory diseases [338]. In vitro and in vivo studies have demonstrated that PTE can inhibit the growth of tumour cells and induce apoptosis by affecting several signalling pathways, such as the PI3K/Akt, MAPK, and NFκB pathways [340,341,342]. Leukaemia cells undergo apoptosis via the MAPK pathway [341].

Lung cancer has been associated with increased cyclooxygenase-2 (COX-2) activity, and PTE has been shown to control NSCLC cell proliferation and apoptosis by targeting COX-2 [343]. By altering the PTEN/Akt pathway, PTE prevents prostate cancer cells from proliferating [344]. Furthermore, PTE works by increasing the tumour suppressor gene PTEN’s acetylation and reactivation. This effect is achieved by suppressing the MTA1/HDAC complex, which usually deacetylates proteins, thereby altering their function. PTE maintains PTEN activity by blocking this complex, which is crucial for controlling the Akt pathway. The Akt pathway is involved in cell growth and survival, and its overactivity can lead to cancer. Therefore, Pterostilbene’s capacity to reactivate PTEN and control the Akt pathway shows its promise as a therapeutic agent in cancer treatment [338]. PTE contributes to the anticancer effect of endoplasmic reticulum stress (ERS) in human oesophageal cancer cells by inducing the ROS-mitochondrial-dependent apoptosis mechanism, which inhibits cell adhesion, invasion, and proliferation [345]. Additionally, recent studies have revealed that PTE improves the sensitivity of triple-negative breast cancer cells to TRAIL-driven apoptosis by triggering the ROS/ERS signalling pathway and boosting DR4 and DR5 expression [346]. It exhibits anticancer effectiveness by inducing autophagy; thus, it may be a promising anticancer drug, as highlighted by its safety profile [347]. Additionally, PTE has been reported to reduce mitochondrial membrane potential, induce cell apoptosis, and suppress cancer cell proliferation in a dose-dependent manner [348,349]. Additionally, PTE showed a dose-dependent suppression of cancer stem cell gene expression and self-renewal capabilities in lung cancer cells co-cultured with M2-TAMs (tumour-associated macrophages with the M2 phenotype). According to Huang et al., this impact seems to be mediated by the downregulation of the cancer-promoting gene MUC1, which inhibits polarisation towards M2 and reduces the accumulation of cancer stem cells (CSCs) [350]. RSV and PTE can target CSCs in a variety of cancers, such as breast cancer colorectal cancer, via a variety of signalling pathways [351].

Furthermore, when combined with sunitinib (SUN), PTE exhibits synergistic antitumor activity against gastric cancer (GC) cell lines [352]. SUN causes mitochondrial iron (II) (mtFe) deposition by suppressing the expression of PDZ domain-containing 8 (PDZD8) [353]. As an iron–sulfur complex, mitochondrial iron is crucial for energy production and other functions; however, it also contributes to cell death through ferroptosis [354]. Inhibiting the recruitment of the iron transporters mtNEET and ABCB8 to PDZD8 by DOX in combination with PTE led to the accumulation of mtFe and an increase in mitochondrial ROS generation, which in turn activated HIF1α, further inducing ER stress and apoptosis [355]. The mechanisms include activation of c-Jun N-terminal kinase and p38 pathways [356,357] with elevated BAX and lower BCL2 expression levels [358,359].

Pterostilbene exhibits greater absorption and corresponding plasma levels than its parent compound, resveratrol [360]. Its anticancer effect has been demonstrated to surpass that of resveratrol in vivo [361], and its additional methyl groups reduce its susceptibility to conjugation metabolism [362]. Through a unique mechanism, trans-3,5,4′-trimethoxystilbene promotes cell cycle arrest and apoptosis with improved potency [363,364], with IC50 values 100–200 times lower than those of resveratrol. Greater pharmacokinetic and tumour-suppressive qualities are possessed by trans-3,4,5,4′-tetramethoxystilbene (DMU212/TMS), and its metabolite has demonstrated increased preclinical cytotoxicity in prostate and ovarian cancers [365]. Moreover, trans-2,4,3′,4′,5′-pentamethoxystilbene was more effective than resveratrol in preventing the growth of breast [366] cancer cells.

Piceatannol, the hydroxylated counterpart, demonstrated direct pro-apoptotic, antimetastatic [367], and tyrosine kinase-inhibiting properties [368] along with equipotency for anti-inflammatory, immunomodulatory, and antiproliferative effects [367].

Lowering the levels of circulating cancer biomarkers, such as insulin-like growth factor 1 and insulin-like growth factor-binding protein 3, was detected [334]. Administration of 0.5 g and 1.0 g of resveratrol resulted in significant inhibition of colorectal cancer cell proliferation [369]. Furthermore, elevated caspase-3 levels were recorded in a phase I clinical trial of hepatic malignancy after administration of resveratrol [370]. A trial of the derivative resveratrol-triphosphatase demonstrated that the compound evoked a better reduction in oxidative stress in obese participants [371].

### 5.5. Lignans

**Figure 7:**
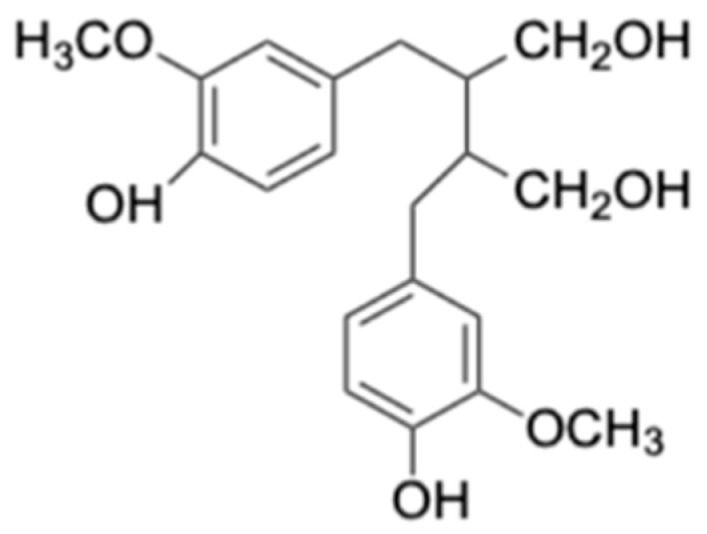
Chemical structure of lignans.

The genus Schisandra (family Schisandraceae) contains a significant group of chemicals known as dibenzocyclooctadiene lignans (DBCLS) (Figure 7), which are distinguished by their distinct chemical structures and wide range of biological activities. This category comprises 40 identified secondary metabolites with hepatoprotective and hepatoregenerative properties, including gomisin A, schisandrin B, schisandrin C, and ʤ-schisandrin. Additionally, it has been confirmed that deoxyschisandrin and ʤ-schisandrin have antiviral properties, that schisandrin and schisandrin B have antioxidant properties, and that schisandrin C and gomisin A have anti-inflammatory properties. Recent studies have focused on investigating DBCLS’s antitumor capability and function in preventing the proliferation of cancer cells [31].

The ability of DBCLS to trigger apoptosis is a key characteristic of its anticancer potential. DBCLS can cause apoptosis in a variety of cancer cell lines, mostly via the mitochondrial apoptotic pathway, according to preclinical research. This entails caspase activation and cytochrome c (cyt-c) release. In addition to their role in apoptosis, DBCLS have cytotoxic effects, including preventing the invasion, migration, and proliferation of cancer cells [372,373,374]. In GBC-SD and NOZ gallbladder cancer cell lines, schisandrin B was able to effectively inhibit the viability and proliferation of cancer cells and induce apoptosis. Moreover, in vivo investigation conducted on nude mice with subcutaneously placed NOZ tumour xenografts revealed that schisandrin B induced apoptosis in cancer cells by controlling the expression of proteins linked to apoptosis [373]. Furthermore, DBCLS can induce oxidative stress in cancer cells by boosting the generation of ROS. Cell death can result from excessive ROS damage to lipids, proteins, DNA, and other biological macromolecules [31]. By controlling the intracellular generation of reactive oxygen species and blocking NADPH oxidase, gamisin L1 exerts potent cytotoxic effects on cancer cells, inducing apoptosis in A2780 and SKOV3 ovarian cancer cell lines [374]. Additionally, gomisin J was reported to have a unique capacity to alter autophagy in cancer cells, especially in MDA-MB-231 and MCF7 cell lines. It initially causes autophagy in a survival form, but after more exposure, autophagy-mediated cell death takes over. The mTOR pathway, which is a component of the PI3K/Akt/mTOR signalling axis and is frequently used by cancer cells to develop drug resistance, appears to be inhibited in this result. Consequently, gomisin J is a promising therapeutic agent, particularly for the treatment of malignancies that have become resistant to standard therapies [375].

In contrast, DBCLS compounds are able to cause cell cycle arrest, especially during the G1/S and G2/M phases, which inhibits the proliferation of cancer cells [31]. Li et al. used in vitro studies on gastric cancer cell lines to investigate the anticancer mechanism of the combination of schisandrin B and the cytotoxic medication apatinib. This synergistic combination arrested the cell cycle in the G0/G1 phase, which inhibited the division of cancer cells. Schisandrin B enhances cytotoxic drug-induced apoptosis of cancer cells and boost the invasion and migration of apatinib in cancer cells [376]. In melanoma cell lines, gomisin A influenced cell cycle arrest and inhibited cell proliferation. Furthermore, it has been demonstrated that gomisin A decreased melanoma cell viability via blocking the cell cycle, and inhibition of cyclin D1, AMPK, ERK, and JNK phosphorylation which is followed by cell cycle arrest in the G0/G1 phase. Additionally, it has been shown to decrease cell invasion and migration and exert antiproliferative effects. Moreover, gomisin A suppresses epithelial-mesenchymal transition, preventing lung metastasis [377]. Together with TNF-α, this compound also inhibited the activity of signal transducer and transcription activation 1 (STAT1) [378].

Gomisin G suppressed the proliferation of MDA-MB-231 and MDA-MB-468 breast cancer cell lines. Gomisin G’s mode of action was predicated on a very significant inhibitory activity on AKT phosphorylation, as well as a reduction in the quantity of phosphorylated retinoblastoma tumour and retinoblastoma tumour suppressor protein. Gomisin G’s activity was also centred on reducing the quantity of cyclin D1 and stopping the cell cycle during the G1 phase [379]. By inducing cell cycle arrest in the G2/M phase, causing tumour cell death, and preventing cancer cell trafficking, Schisantherin A showed a strong antiproliferative effect on MKN45 and SGC-7901 gastric cancer cell lines. Furthermore, schisantherin A increases the generation of reactive oxygen species, which are necessary for JNK phosphorylation [380]. Due to their capacity to alter matrix metalloproteinase (MMP) activity, which is essential for cancer metastasis, several studies have indicated that DBCLS compounds can prevent cancer cell migration and invasion. By boosting the number of heat shock proteins, schisandrin B also prevents the development, migration, and invasion of cancer cells (c). In the MDA-MB-435S breast cancer cell line, schisandrin B (encapsulated in liposomes) combined with PFV-modified epirubicin cytotoxic medication increased cytotoxicity, influenced the formation of vascular mimicry, and prevented tumour invasion and spread. The control of vimentin, E-cadherin, vascular endothelial growth factor (VEGF), and matrix metalloproteinase-9 (MMP-9) expression forms the basis of the mechanism of action against cancer cells. Mice have also been used in tests that showed an increase in cancer cell apoptosis [381]. Additionally, schisandrin B completely prevented the growth, proliferation, and invasion of gastric cancer cells in both in vitro and in vivo experiments. Schisandrin B was also shown to suppress STAT3 phosphorylation, induces apoptosis, and boost the effectiveness of 5-fuorouracil [382]. Schisandrin B was shown to successfully suppress cell migration, invasion, and multiplication in both in vitro and in vivo investigations. It has also been reported to increase apoptosis in MG63, Saos2, and U2OS osteosarcoma cell lines. Additionally, studies on healthy cells have demonstrated that schisandrin B has no detrimental effect on the viability of these cells [383].

The ability of lignans to induce oxidative stress in cancer cells may be the cause of schisandrin B’s ability to inhibit the proliferation and induce apoptosis of DU145 and LNCaP prostate cancer cell lines. It also inhibits the cell cycle in the S phase [384]. Moreover, schisandrin B inhibits cell division, migration, and invasion, according to in vivo experiments conducted on animal models. Schisandrin B also induces apoptosis by inhibiting the PI3K/Akt and Wnt/β-catenin signalling pathways, although it has no detrimental effects on healthy cells [383]. In order to investigate the action against BGC-823 human gastric cancer cells, Li et al. employed liposomes to encapsulate the cytotoxic medication vinoreblin, R8 peptide, and schisandrin B. Schisandrin B was utilised to suppress metastasis, vinoreblin was employed as a chemotherapeutic drug, and peptide R8 was utilised for its ability to increase cellular absorption. The liposomal complex inhibited invasion and metastasis by lowering the levels of VE-Cadherin, PI3K, VEGF, HIF-1α, FAK, and MMP-2, thereby inducing apoptosis in cancer cells. Furthermore, in vivo experiments have demonstrated that liposomes specifically gather in cancer cell-occupied areas and cause cell death [385].

Breast and ovarian cancer cell lines were used to assess the ability of schisandrin B to treat doxorubicin-resistant malignancies. According to previous studies, schisandrin B significantly increases the intracellular accumulation of doxorubicin by inhibiting the expression and activity of P-glycoprotein. Schisandrin B also decreased the expression of the anti-apoptotic protein survivin [386]. Additionally, schisandrin B and docetaxel together decreased the viability of Caski cervical cancer cells, prevented colony formation, induced apoptosis, and prevented the invasion of tumour cells. Furthermore, BALB/c nude mice xenografted with Caski cells were used for in vivo investigations. These outcomes demonstrate this combination’s synergistic effect [387]. Despite the fact that DBCLS from the Schisandra genus have demonstrated encouraging anticancer properties in both in vitro and in vivo models, no research has progressed to clinical trials. This limits the comprehension of the safety, dose, and therapeutic effectiveness of lignans in humans. Furthermore, a thorough knowledge of the metabolism, distribution, excretion, and mode of action of these lignans in the human body is currently lacking, which necessitates further research [31].

## 6. Conclusions and Future Directions

New approaches to cancer prevention and treatment have been made possible by natural polyphenols that have drawn a lot of interest in response to the increasing demand for the creation of novel therapeutic and preventive solutions derived from natural sources. Compared to the previous literature, this review offers a broader scope by examining not only individual polyphenol classes but also their dietary sources, bioavailability, and recent advances in nanoformulation strategies in clinical settings. By integrating mechanistic insights with translational findings, this review underscores the therapeutic potential of polyphenols in cancer management. These findings support ongoing efforts to incorporate natural compounds into cancer prevention and treatment strategies, offering a safer and potentially cost-effective alternative to conventional therapies.

Although the molecular mechanisms underlying their wide range of biological activities are not completely understood, they are likely linked to cell cycle disruption, apoptosis induction, and improved the efficacy of currently available cytotoxic medications. In recent years, many studies have examined the antiproliferative effects of polyphenols on a variety of malignant tumours, both in vivo and in vitro, while polyphenols causing normal cells little to no harm. Furthermore, natural polyphenols enhance the therapeutic effectiveness of current treatments and reduce the adverse effects associated with chemotherapy. Thus, the promising potential of natural polyphenols makes them appealing candidates for future oncological applications. Despite phase I and II clinical trials, polyphenols have shown their critical importance in cancer prevention; however, further clinical research is still needed to completely understand the effectiveness of natural polyphenols in cancer treatment. The journey from the laboratory to the clinic remains essential. Future efforts should focus on human clinical studies to assess the safety and efficacy of these compounds and close the large gap between preclinical discoveries and clinical applications.

## Abbreviations

ABCB8	ATP-Binding Cassette subfamily B member 8
ADAM17	A Disintegrin And Metalloprotease 17
ADP	Adenosine Diphosphate
AgNPs	Silver nanoparticles
Ahr	Aryl hydrocarbon receptor
AIF	Apoptosis Inducing Factor
ALP	Alkaline Phosphatase
ALT	Alanine Transaminase
Antioxcin6	Mitochondrial Targeted Antioxidant
AP-1	Activator Protein-1
APAF1	Apoptotic Protease-Activating Factor 1
ASK-1	Apoptosis signal-regulating kinase 1
AST	Aspartate aminotransferase
ATL	Adult T Cell Leukaemia
ATP	Adenosine Triphosphate
BAX	Bcl-2 Associated X Protein
B-CLL	B-cell Chronic Lymphocytic Leukaemia
Bcl-2	B-Cell Lymphoma 2
BNIP3	BCL-2 Interacting Protein 3
CA	Caffeic Acid
CASP	Caspase
CAT	Catalase
CCND1	Cyclin D1
CD44	Cell surface adhesion receptor
CDC2	Cell division cycle protein 2
CDH1	Cadherin 1
CDH2	Cadherin 2
CDK1	Cyclin-Dependent Kinase 1
CDK2	Cyclin-Dependent Kinase 2
CDK4	Cyclin-Dependent Kinase 4
CDK1A	Cyclin-Dependent Kinase Inhibitor 1A
CDK1B	Cyclin-Dependent Kinase Inhibitor 1B
CEA	Carcinoembryonic Antigen
C/EBP	CCAAT/Enhancer Binding Protein
c-FLIP	Cellular FLICE (FADD-like IL-1β-converting enzyme)-Inhibitory Protein
cIAP1	Cellular Inhibitor of Apoptosis Protein-1
cIAP2	Cellular Inhibitor of Apoptosis Protein-2
CIS	Cisplatin
CIP2A	Carcinogenic Inhibitor of Protein Phosphatase 2A
CLNs	Curcumin-containing catanionic Lipid Nanosystems
Col1α1	Collagen, type I, alpha 1
Col3α1	Collagen type III alpha 1 chain
ConA	Concanavalin A
COX-2	Cyclooxygenase-2
CSCs	Cancer Stem Cells
CTNNB	Catenin Beta 1
CUR	Curcumin
CVD	Cardiovascular Disease
CYCS	Cytochrome C, Somatic
CYP1A1	Cytochrome P450, family 1, subfamily A, Polypeptide 1
CYP3A4	Cytochrome P450 family 3, subfamily A, polypeptide 4
Cyt-c	Cytochrome complex
DBCLS	Dibenzocyclooctadiene lignans
DENA	Diethylnitrosamine
DLC1	DLC1 Rho GTPase Activating Protein
DMBA	7,12-Dimethylbenz[a]anthracene
DMBS	3,4-Dimethoxybenzenesulfonamide
DMH	1,2-Dimethylhydrazine
DMSO	Dimethylsulfoxide
DNA	Deoxyribonucleic Acid
DOT1L	Disruptor of Telomeric Silencing 1-Like
DOX	Doxorubicin
DR4	Death receptor 4
DR5	Death receptor 5
DSS	Dextran Sulfate Sodium
DUSP1	Dual specificity protein phosphatase 1
DUSP3	Dual specificity protein phosphatase 3
DUSP5	Dual specificity protein phosphatase 5
*D. caffra*	*Dovyalis caffra*
EAFE	Ethyl Acetate Fractionated Extract
ECM	Extracellular Matrix
EGCG	Epigallocatechin-3-Gallate
EGF	Epidermal Growth Factor
EGFR	Epidermal Growth Factor Receptor
EMT	Epithelial-Mesenchymal Transition
EPI	Epicatechin
ER	Endoplasmic Reticulum
ER	Estrogen Receptor
α-ER	Estrogen Receptor Alpha
β-ER	Estrogen Receptor Beta
ERK	Extracellular Signal-Regulated Proteinase
ERS	Endoplasmic Reticulum Stress
EZH2	Enhancer of Zeste Homologue 2
FAD	Flavin Adenine Dinucleotide
FAK	Focal Adhesion Kinase
FasL	Fas Ligand
FIH-1	Factor-Inhibiting HIF-1
FN	Fibronectin
FOXM1	Forkhead Box M1
5-FU	5-Fluorouracil
GA	Gallic Acid
GADD45A	Growth Inhibition And DNA-Damage-Inducible 45 Alpha
GADD45B	Growth Arrest and DNA-Damage Inducible Beta
GC	Gastric Cancer
Gli-1	Glioma-associated oncogene
GSK3β	Glycogen Synthase Kinase-3 beta
GPx	Glutathione Peroxidase
GPx4	Glutathione Peroxidase 4
GRP78	78 kDa Glucose-Regulated Protein
GR	Glutathione Reductase
GSH	Glutathione
HA	Hyaluronic Acid
HC	Hepatic Cells
HCC	Hepatocellular Carcinoma
HDAC	Histone Deacetylase
HEPG2	Human Hepatocellular Liver Cancer Cells
HER2	Human Epidermal Growth Factor Receptor 2
HER2-TK	Human Epidermal Growth Factor Receptor 2-Tyrosine Kinase
HETNPs	Hesperetin-loaded Nanoparticles
HGF	Hepatocyte Growth Factor
HIF-α	Hypoxia-Inducible Factor 1-Alpha
HO-1	Heme Oxygenase-1
Hrk	HaraKiri
HSCs	Hepatic Stellate cells
HSP	Hesperetin
HTLV-1	Human T-Lymphotropic Virus Type 1
HUVEC	Human Umbilical Vein Endothelial Cells
Hyp	Hydroxyproline
IC50:	Half maximal inhibitory concentration
IGF1R	Type 1 Insulin-Like Growth Factor Receptor
IgG	Immunoglobulin G
IgM	Immunoglobulin M
IKKβ	Inhibitory kappa B Kinase Beta
IL-6	Interleukin 6
IL-10	Interleukin 10
IL-12	Interleukin 12
IRAK4	Interleukin 1 Receptor Associated Kinase 4
IR-beta	Insulin Receptor-Beta Subunit
IUPAC	International Union of Pure and Applied Chemistry
JAK	Janus Kinase
JNK	Jun N-Terminal Kinases
LC3B	Light chain 3B
LPO	Lipid Peroxidation
67LR	67 kDa Laminin Receptor
MAPK	Mitogen-Activated Protein Kinase
MARK4	Microtubule Affinity-Regulating Kinase 4
MBS	4-Methoxybenzenesulfon
MD-2	Myeloid Differentiation Factor 2
MCL-1	Myeloid Cell Leukaemia-1
MCP-1	Monocyte Chemoattractant Protein-1
MDM2	Mouse Double Minute 2 Homologue
MDR	MultiDrug Resistance
MEK	Mitogen-Activated protein kinase Kinse
MiR	MicroRNA
MMPBSA	Molecular Mechanics Poisson-Boltzmann Surface Area
MMP-2	Matrix Metalloproteinase-2
MMP-9	Matrix Metalloproteinase-9
MMP	Matrix Metalloproteinase
MM	Multiple myeloma
MPA	Medroxy Progesterone Acetate
mRNA	Messenger ribonucleic acid
MT1A	Metallothionein 1A
MT1F	Metallothionein 1F
mtFe	Mitochondrial iron (II)
mtNEET	Mitochondrial protein mitoNEET
MTX	Methotrexate
mTOR	Mammalian Target Of Rapamycin
M2-TAMs	Tumour-Associated Macrophages with the M2 phenotype
NAD	Nicotinamide Adenine Dinucleotide
NADPH	Reduced Nicotinamide Adenine Dinucleotide Phosphate
Nar	Naringenin
NFKBIA	Nuclear Factor-Kappa-B-Inhibitor Alpha
NF-Kb	Nuclear Factor Kappa B
NLRP3	NLR Family Pyrin Domain-containing 3
Nrf2	Nuclear Factor Erythroid Related Factor 2
NSCLC	Non-Small Cell Lung Carcinoma
OS	Oxidative Stress
P-AKT	Phospho-Akt
PAI	Plasminogen Activator Inhibitor-1
PARP-1	Poly-ADP-Ribose Polymerase 1
PCNA	Proliferating Cell Nuclear Antigen
PC-3	Prostate Cancer-3
PBMC	Peripheral Blood Mononuclear Cells
PDAC	Pancreatic Ductal Adenocarcinoma
PDZD8	PDZ domain-containing 8
PEGFR	Phosphorylated-epidermal growth factor receptor
PEL	Primary Effusion Lymphoma
PERK	Phosphorylation of Extracellular Signal-Related Kinase
PGE2	Prostaglandin E2
P-gp	P-glycoprotein
PhK	Phosphorylase Kinase
PI3K	Phosphatidylinositol 3-kinase
PKA	Protein Kinase A
PKB	Protein Kinase B
PKM2	Pyruvate Kinase Isozymes M1/M2
PLGA	Poly (Lactic-co-Glycolic Acid)
PMA	Phorbol Myristate Acetate
PPARG	Peroxisome Proliferator-Activated Receptor Gamma
PRKCA	Protein Kinase C Alpha Gene
Prp4B	Pre-RNA processing factor 4B
PSA	Prostate-Specific Antigen
PTEN	Phosphatase and Tensin Homologue
PTE	Pterostilbene
PTX	Paclitaxel
RA	Rosmarinic Acid
RANK	Receptor Activator of Nuclear Factor Kappa-Β
RANKL	Receptor Activator of Nuclear Factor Kappa-Β Ligand
RASSF-1a	Ras-Association Domain Family-1a
Rb	Retinoblastoma protein
RNA	Ribonucleic Acid
ROS	Reactive Oxygen Species
RSV	Resveratrol
SA	Sinapic Acid
SaNPs	Sinapic acid-loaded Nanoparticles
SCC	Squamous Cell Carcinoma
SCCHN	Squamous Cell Carcinoma of The Head and Neck
SCID	Severe Combined Immunodeficiency
Shh	Sonic hedgehog protein
SIRT1	Sirtuin 1
SKP2	S Phase Kinase Associated Protein 2
SLC7A11	Solute Carrier Family 7 Member 11
SLNs	Solid Lipid Nanoparticles
SOD	Superoxide Dismutase
SPRR2D	Small Proline Rich Protein 2D
STAT	Signal Transducer and Activator of Transcription
Sufu	Suppressor of Fused Protein
t-BHP	tert-Butyl Hydroperoxide
TGF-β1	Transforming Growth Factor Beta 1
TIMP-1	Tissue Inhibitor of Metalloproteinases 1
TIMP-2	Tissue Inhibitor of Metalloproteinases 2
TLR4	Toll-Like Receptor 4
TMBS	3,4,5-Trimethoxybenzenesulfonamide
TME	Tumour Microenvironment
TNBS	2,4,6-Trinitrobenzene Sulfonic Acid
TNFRSF11B	TNF Superfamily 11B Receptor
TNFRSF25	Tumour Necrosis Factor Receptor Superfamily 25
TNF-α	Tumour Nerosis Factor-Alpha
TOPK	T-LAK Cell-Originated Protein Kinase
TRAIL	Tumour Necrosis Factor (TNF)- Related Apoptosis-Inducing Ligand
TRIF	TIR-Domain-Containing Adapter-Inducing Interferon-β
UGT1A3	UDP Glucuronosyltransferase Family 1 Member A3
UPA	U-Plasminogen Activator
UV	Ultraviolet rays
UVB	Ultraviolet rays type B
VCAM-1	Vascular Cell Adhesion Molecule-1
VEGF	Vascular Endothelial Growth Factor
VV-IL-24	Vaccinia Virus that encoded the IL-24 gene
WHO	World Health Organization
Wnt	Wingless-Related Integration Site
WT1	Wilms Tumour Protein
XIAP	X-Linked Inhibitor of Apoptosis
α-SMA	Alpha-Smooth Muscle Actin
γCD	Gamma-Cyclodextrin
